# Newton’s cradle-like allosteric mechanism explains regulatory RsmE RNA binding

**DOI:** 10.1038/s41467-026-72126-z

**Published:** 2026-04-22

**Authors:** Esteban Finol, Fred F. Damberger, Miroslav Krepl, Timo Flügel, Priscilla Dietrich, Thomas C. T. Michaels, Beat Vögeli, Jiří Šponer, Frédéric H-T. Allain

**Affiliations:** 1 Institute for Biochemistry, Department of Biology, ETH Zurich, Zurich, Switzerland; 2 Institute of Biophysics of the Czech Academy of Sciences, Brno, Czechia; 3 Department of Biochemistry and Molecular Genetics, University of Colorado Anschutz Medical Campus, Aurora, CO USA

**Keywords:** Solution-state NMR, Molecular modelling, Computational biophysics, RNA, Molecular conformation

## Abstract

In the bacterial Csr/Rsm system, non-coding RNAs activate mRNA translation by removing homodimeric Csr/Rsm proteins from ribosome-binding sites of mRNAs. In *Pseudomonas protegens*, each RsmZ ncRNA sequesters up to five RsmE dimers sequentially and specifically within a narrow affinity range, functioning as a ‘protein sponge’. Although the RsmE binding cascade is cooperative, binding of the highest affinity stem-loop RNA in RsmZ (SL2) reduces RNA binding affinity at the second site 10- to 30-fold. This unusual negative cooperativity may facilitate RsmE release from tightly bound mRNA for handover to the non-coding RNA, yet the underlying mechanisms remain unclear. Using Isothermal Titration Calorimetry, NMR spectroscopy and Molecular Dynamics, we reveal an allosteric mechanism resembling a Newton’s cradle, coupling the binding at one site to conformational and dynamic changes at the second site, explaining the reduced affinity of the second binding event, and handover of RsmE dimer from mRNA to the ncRNA RsmZ.

## Abstract (web summary)

RsmZ acts as a “protein sponge” by stripping RsmE off mRNAs to restore translation. This study reveals why binding at one RsmE site weakens the other via a Newton’s cradle-like allostery, enabling efficient handover of RsmE from mRNAs to the regulatory RNA.

## Introduction

In Gammaproteobacteria such as *Salmonella*, *Pseudomonas*, *Escherichia*, *Erwinia* and *Vibrio*, homodimeric proteins from the carbon storage regulator/repressor of secondary metabolites (Csr/Rsm) family bind the Shine-Dalgarno (SD) sequence in the 5′ untranslated region of messenger RNA (mRNA). As intertwined dimers, they employ one of their two RNA-binding sites to recognise the guanine-guanine-adenine (GGA) motif in the SD sequence while the other binding site engages a second GGA motif in the mRNA^1^. Their high affinity for the SD allows Csr/Rsm protein dimers to compete with the small (30S) subunit of the ribosome, thereby repressing translation initiation of hundreds of mRNAs^2^. Consequently, Csr/Rsm proteins serve a global regulatory system of gene expression for many fundamental cellular processes. They repress biofilm formation, quorum sensing, gluconeogenesis and glycogen metabolism, and they activate virulence, glycolysis, cell motility and pathogenesis^3,4^.

In response to environmental stimuli, these bacteria express small non-coding RNA (ncRNA) that can sequester and remove Csr/Rsm-type proteins from mRNA, allowing for their translation^5^. In the *Pseudomonas protegens* CHA0 strain (formerly *P. fluorescens*), each 127-nucleotide RsmZ ncRNA molecule seizes four RsmE dimers, making use of eight GGA motifs: four located in the loops of stem-loop (SL) RNA and four in single-strand regions^6^. In isolation, shorter RNA molecules containing the individual GGA motifs bind RsmE dimers with dissociation constants (*K*_D_) in the μM range^7^. The only exception is the GGA motif within SL2 in RsmZ ncRNA, which binds RsmE at very high affinity (*K*_D_ in the low nM range). Since all isolated binding sites have micromolar or lower dissociation constants, RsmZ sequesters RsmE dimers in a sequential and cooperative manner^8^. We showed that the RsmE homodimer exhibits a negative cooperativity when the first SL RNA bound has a high (nM) affinity. The second binding event has an order of magnitude lower affinity, and we proposed that this furnished the non-coding RNA RsmZ with a means to displace RsmE from the SD site of mRNAs.

Here we investigate the origin of this negative allostery using isothermal calorimetry (ITC), NMR spectroscopy and molecular dynamics simulations. We show that the binding of a high-affinity SL RNA induces both conformational and dynamic changes in the empty site, resulting in increased conformational entropy. This creates both an enthalpic and entropic penalty for the binding of a second SL RNA. We also investigate the structural and dynamic origins of this allosteric communication between the binding sites. The RsmE dimer acts like Newton’s cradle to transmit conformational entropy between binding sites so that the ncRNA RsmZ can displace RsmE from mRNAs to allow their transcription.

## Results

**Fig. 1:**
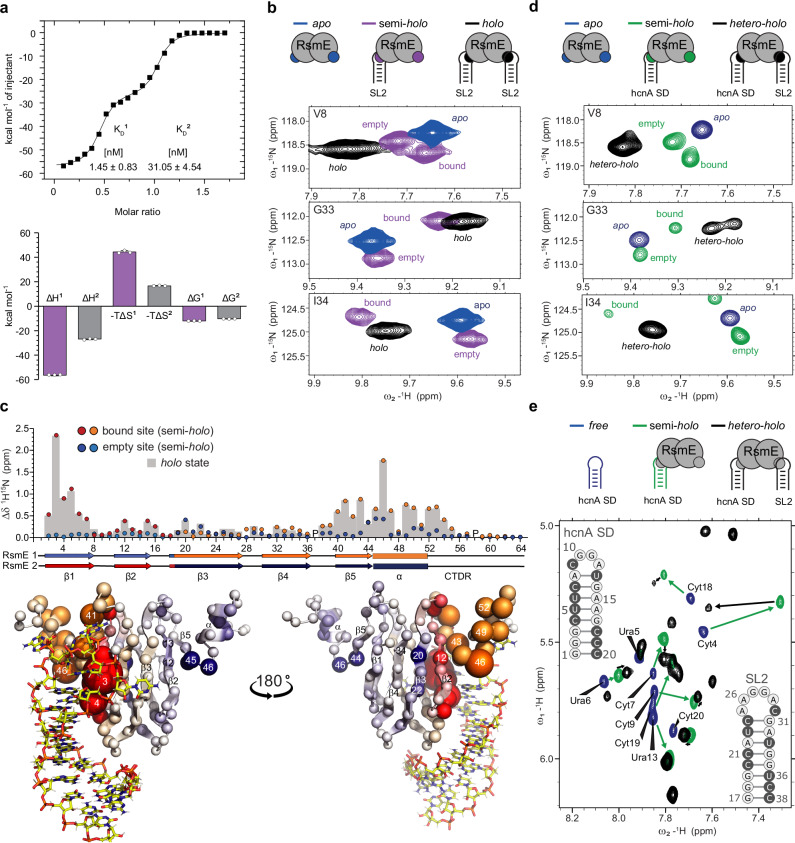
Negative allostery with two distinct intermediate states in RsmE dimer. **a** Isothermal titration calorimetry (ITC)-derived binding curve of SL2 RNA titrated to the RsmE dimer. Molar ratio corresponds to the RNA:RsmE monomeric ratio. The bar-plot shows thermodynamic changes for the first and second binding events. Values, found in Supplementary Table 1 are derived from the average of three independent ITC titrations (shown data points), and errors are obtained from their standard deviation. **b** Three backbone amide peaks in overlaid ^1^H^15^N-HSQC spectra of *apo* (blue), semi-*holo* (purple) and *holo* (black) SL2-^15^N-RsmE dimer states. **c** NH-CSPs in RsmE dimer upon binding one SL2 RNA. NH-CSPs shown for bound and empty sites. Binding sites are composed of residues from both RsmE monomers: dots for NH-CSPs in the semi-*holo* state are differently colour-coded for the two monomers (light and dark colours) and the two binding sites (blue and red/orange). The underlying grey bar plot shows NH-CSPs of the *holo* state. P indicates proline residues. NH-CSPs are shown on a structural model of the semi-*holo* state, with NH-CSPs of bound and empty sites indicated by coloured spheres. Residues are colour-coded according to the secondary structure diagram below the NH-CS plots in (**c**). Intensity of colours and diameters of spheres indicate the magnitude of NH-CSP at every residue. The structure model is derived from the *holo* SL2-RsmE dimer complex (PDB:2MFE) with one SL2 RNA removed. **d** Three backbone amide peaks in overlaid ^1^H^15^N-HSQC spectra of *apo* (blue), semi-*holo* (hcnA SD-^15^N-RsmE dimer in green) and *hetero-holo* (black) hcnA SD-^15^N-RsmE dimer-SL2 states. **e** H5–H6 region of 2D ^1^H^1^H-TOCSYs. H5–H6 assignment of the 9 pyrimidines in the hcnA SD RNA in its free semi-*holo* and *hetero-holo* states are indicated. H5–H6 peaks of the SL2 RNA are assigned in Supplementary Fig. 1d. Arrows indicate the CS changes between RsmE dimer states. Secondary structures for SD and SL2 RNA are shown.

As shown previously^8^ and repeated here (Fig. 1), titration of RsmE dimer with isolated SL2-RNA exhibits a 10- to 30-fold reduced affinity for the second RNA binding (see Supplementary Table 1). This represents an example of anti-cooperativity. Moreover, examination of the backbone amide (NH) cross peaks of RsmE upon binding to only one SL2 RNA revealed the presence of two signals from the singly bound (semi-*holo*) state whose shifts are distinct from both unbound (*apo*) and doubly bound (*holo*) dimeric protein (Fig. 1b). For each amide displaying these two signals one could generally be identified with the bound site and the other with the empty site, and thus clearly reported on the broken symmetry due to the singly bound RsmE homodimer. Although the backbone NH chemical shift perturbations (NH-CSPs) of the bound site were generally similar in magnitude to those of the *holo* dimer (Fig. 1c), the distinguishable shift positions of the singly bound versus doubly bound states suggest that the strong RNA binding to the first binding site is different from the doubly bound *holo* state. In addition, shift changes at the empty site suggest that RNA binding to the first site allosterically alters the conformation of the second, empty RNA binding site. These observations were reproduced using a different high affinity-binder a SL RNA containing the SD sequence from the hcnA mRNA, henceforth termed SD RNA (Supplementary Note 1 and Supplementary Fig. 1). Binding of SL2 RNA from RsmZ to a 1:1 SD-bound RsmE dimer (semi-*holo*) led to chemical shift changes in both the SD RNA and the SD-bound site of the RsmE dimer (Fig. 1d, e), confirming that SL2 binding induces changes in the SD-bound site and suggesting that anti-cooperativity in the RsmE dimer contributes to derepress RsmE-bound mRNAs. Consistent with this, ITC measurements show that the *K*_D_ of SD RNA binding to *apo* RsmE was ~10 nM, whereas when SL2 RNA was already bound, the *K*_D_ for SD RNA binding to the remaining site was ~300 nM, a 30-fold lower binding affinity (Supplementary Fig. 1a, b).

### Semi-*holo* state has multiple conformations in the bound site

**Fig. 2:**
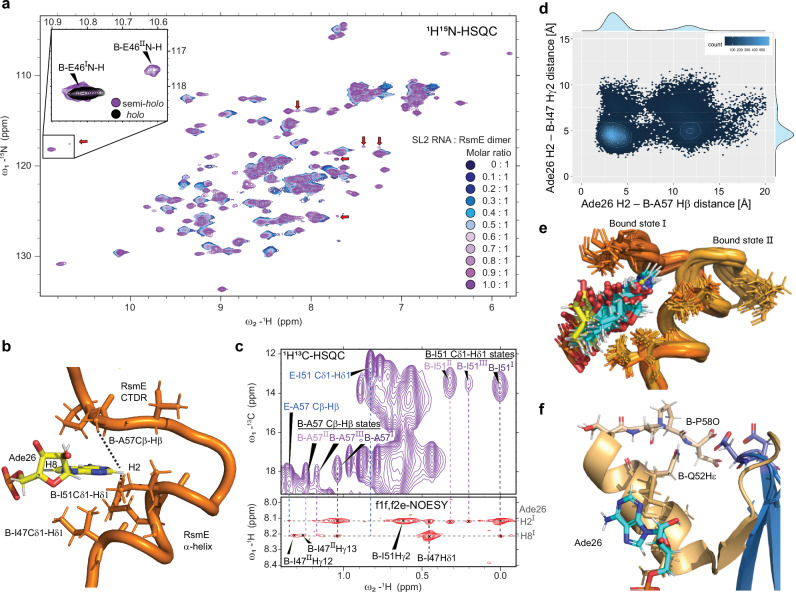
The binding of one SL2 RNA leads to multiple slowly exchanging conformations in the bound α-helix and CTDR of the RsmE dimer. **a** Overlaid ^1^H^15^N-HSQC spectra from the titration of SL2 RNA into ^15^N-RsmE dimer, where only the first binding site was saturated. The insert shows an expanded view of the NH peak from bound glutamate 46 (B-E46) in the ^1^H^15^N-HSQC spectra from semi-*holo* (purple) and *holo* (black) ^15^N-RsmE dimer. The B-E46 state ΙΙ NH peak (B-E46^ΙΙ^) is also labelled. **b** α-helix and CTDR of the RsmE dimer bound to Ade26 as previously determined in solution (PDB: 2MFE). **c** Top: methyl region in the ^1^H^13^C-HSQC of semi-*holo* RsmE dimer. Labels indicate the CS of I51Cδ1-Hδ1 and A57Cβ-Hβ in the empty site (blue), minor states ΙΙ and ΙΙΙ (light purple and purple) and major bound (dark purple) conformations. Bottom: Ade26 H2 and H8 NOE towers in the 2D f1 ^13^C-filtered f2-^13^C edited NOESY spectrum. **d** 2D-plot of correlated atomistic distances in the 10 μs MD simulations (total: 400,000 frames). **e** Structural alignment for 10 representative snapshots of the two abundant MD-derived conformations in the α-helix and CTDR of the semi-*holo* RsmE dimer (state Ι and ΙΙ are coloured in dark and light orange, respectively). Ade26 is shown as coloured sticks (carbons are yellow for state Ι and cyan for state ΙΙ). **f** Contacts observed in the bound state ΙΙ conformation in MD simulations.

In order to understand the origin of negative allostery, we examined in detail the NMR spectra of the RsmE dimer bound to one SL2 RNA. Titration of RsmE dimer with SL2 RNA led to the gradual disappearance of the apo peaks, with a new set of peaks appearing corresponding to the semi-*holo* state. At 1:1, neither *apo* nor *holo* state peaks where detectable, although they would be expected due to the *K*_D1_ and *K*_D2_ values obtained with ITC. This is probably because *K*_D1_ (1.4 nM) is at the lower bound of what can be quantified reliably with ITC. It is likely to be significantly lower than 1 nM, and this would increase the cooperativity, resulting in much smaller populations of the *apo* and *holo* states. Close inspection of the ^1^H^15^N-HSQC spectrum of the semi-*holo* form revealed a set of additional low-intensity backbone amide signals (Fig. 2a), suggesting the existence of a minor conformation in slow exchange with the major form. In the ^1^H^13^C-HSQC, we could even detect three conformations for some side chains (Supplementary Fig. 2a–c). Quantifying the NMR signals in the ^1^H^13^C-HSQC spectrum indicated that the abundance of these distinguishable conformations in slow exchange is approximately 61, 33 and 6%. In what follows, we term these semi-*holo* states: state Ι (or major), ΙΙ (or minor) and ΙΙΙ respectively and due to the limited information obtainable, we consider the chemical shift assignment of state ΙΙΙ as tentative. From the assignments, the regions involved in slow conformational exchange among these states could be mapped to the α-helix and the downstream C-terminal disordered region (CTDR) of the RNA-bound side of the semi-*holo* complex as exemplified by the NMR signals of the bound Isoleucine 47 and Isoleucine 51 in the α-helix and Alanine 57 in the CTDR, respectively (B-I47, B-I51 and B-A57 in Fig. 2b, c, Supplementary Figs. 2, 3 and Supplementary Note 2). For clarity, we indicate bound site and empty site residues with B- and E- respectively. In the previously determined structure of the *holo* complex in solution^7^, amino acids in these regions interact with adenine 26 (Ade26) in the SL2 hexa-loop of RsmZ ncRNA (Fig.2b). The aromatic nature of the adenine base can induce a ring current effect on neighbouring nuclei. Thus, subtle changes in Ade26 base orientation can lead to distinct proton chemical shifts for protein sidechains in proximity. These sidechains in the major (state Ι) conformation of the semi-*holo* complex underwent similar CSPs upon SL2 binding to those observed in the *holo* complex (Supplementary Fig. 2), confirming that this major conformation has a local structure closely similar to the *holo* structure. To probe the RNA-protein contacts in the three semi-*holo* states of SL2-RsmE dimer complex a 2D f1-filtered f2-^13^C edited NOESY experiment was recorded with ^13^C^15^N-labelled protein and unlabelled SL2 RNA. Intermolecular NOEs for the major and minor states could be detected and they were associated with subtle chemical shift differences of Ade26 (H2^Ι^ = 8.116 ppm and H2^ΙΙ^ = 8.121 ppm; H8^Ι^ = 8.215 ppm and H8^ΙΙ^ = 8.221 ppm). Clear intermolecular NOEs between Ade26 H2 and the methyl protons of B-I51 Cδ1 in the bound α-helix for the major (Ι) and both minor conformations confirmed that these interactions persist even in the minor forms (Fig. 2c). In contrast, no intermolecular NOEs were observed between Ade26 H2 and the state ΙΙ of B-A57 in the CTDR (e.g. no Ade26–B-A57^ΙΙ^Hβ NOE in Fig. 2c), suggesting the existence of a distinct CTDR conformation with no Ade26 contacts.

### MD simulations show second bound state without Ade26-CTDR contacts

To gain further spatial and temporal insights into the conformational space explored by the RsmE dimer in the presence of one SL2 RNA, we performed explicit solvent atomistic molecular dynamics (MD) simulations. For this purpose, one SL2 RNA was removed from the previously determined *holo* SL2-RsmE dimer structure. This semi-*holo* structure was then equilibrated, and four 10 μs MD simulations were performed. To track structural changes in the Ade26-RsmE dimer interface, the distances between the Ade26 H atoms and the methyl group H atoms in the SL2-bound α-helix (B-I47Hγ2 and B-I51Hδ1) and CTDR (B-A57Hβ) of the RsmE dimer were measured throughout these simulations. The two-dimensional plots correlating these distances (for instance, Ade26H2–B-I47Hγ2 and Ade26H2–B-A57Hβ distances in Fig. 2d) showed two clear peaks of abundant conformations, suggesting that the CTDR can adopt a second conformation where the Ade26H2–B-A57Hβ distance is greater than 10 Å while the Ade26H2–B-A47Hγ2 distance remained short, indicating Ade26 still interacted with the bound α-helix of RsmE. To characterise this second conformation 20 frames were retrieved from the MD simulations; ten frames from the most abundant peak which is similar to the *holo* structure and ten frames from the second peak containing conformations where the CTDR is not interacting with Ade26 (Fig. 2e). In this second conformation the carbonyl of B-P58 interacts with the sidechain amide group of B-Q52 of the α-helix (Fig. 2f). By tracking the B-Q52Hε–B-P58O atomic distance across the four 10 μs MD simulations (Supplementary Fig. 4a) a single event was detected which showed the emergence of this second conformation, suggesting that the transition represents a rare event on a 10 μs timescale. Three additional 10 μs MD simulations that started from this minor conformation indicated that it is similarly stable on the μs MD timescale (Supplementary Note 3 and Supplementary Fig. 4b). Support that the minor state detected in the MD is related to the minor state identified by NMR is derived from large amide shift differences observed between major and minor forms for the sidechain of B-Q52 and for the backbone of B-T56 (Supplementary Fig. 5). Collectively these observations support the existence of at least two slowly exchanging conformations associated with the loss of Ade26-CTDR contacts in the semi-*holo* SL2-RsmE dimer state.

### β3–β4 loop/RNA stem contacts increase the first binding affinity

**Fig. 3:**
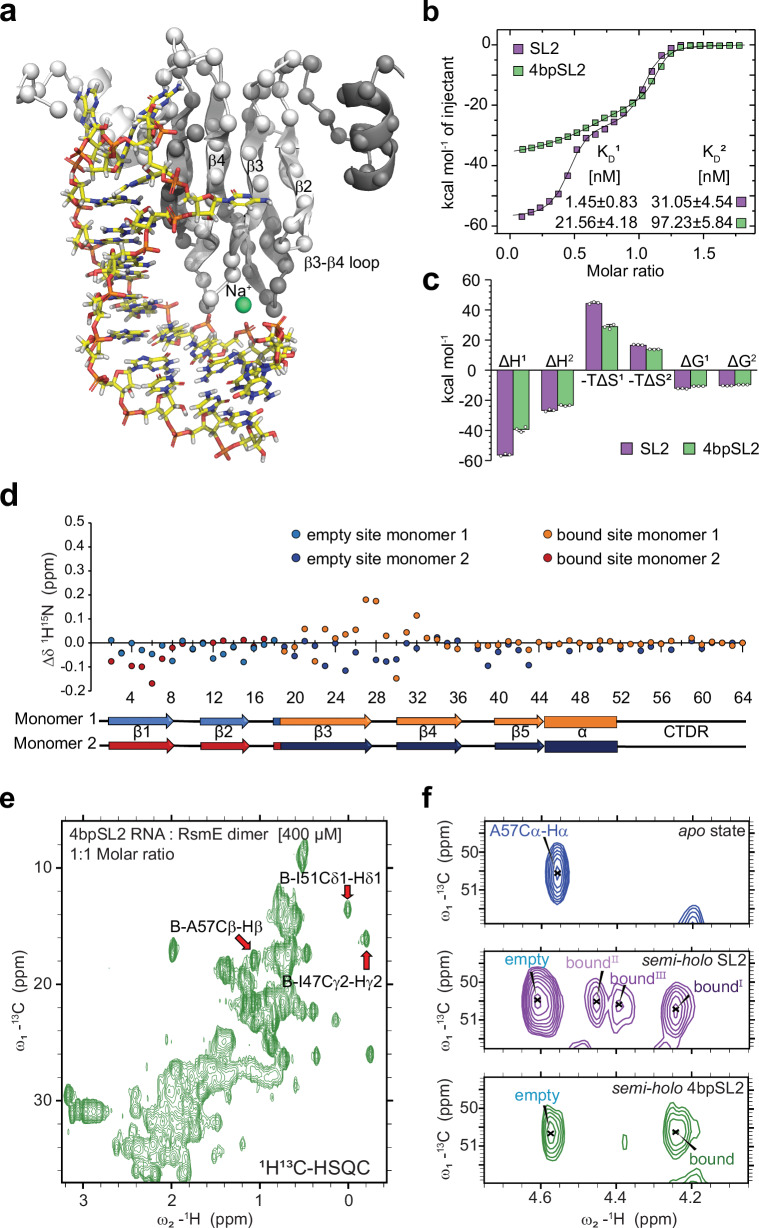
Transient interactions between the RNA stem and the empty β3–β4 loop increase binding affinity and conformational entropy in the semi-*holo* state. **a** Semi-*holo* SL2-RsmE dimer complex from one frame of the MD simulations. Monomers are differently coloured in light and dark grey. The coordinated sodium ion is shown in green. **b** ITC curves for the binding of SL2 and 4bpSL2 RNA to the RsmE dimer. **c** Bar plot with thermodynamic changes for the first and second binding events obtained from ITC. Values found in Supplementary Table 1 are derived from the average of three independent ITC titrations (shown data points), and errors are obtained from their standard deviation. **d** Difference in NH-CSPs between the semi-*holo* SL2- and 4bpSL2-RsmE dimers. **e** The methyl region of the ^1^H^13^C-HSQC spectrum from the semi-*holo* 4bpSL2-RsmE dimer complex (green). Red arrows indicate the chemical shifts where additional minor conformation peaks were observed in the semi-*holo* SL2-RsmE dimer complex. **f** A57 CαHα peaks in different ^1^H^13^C-HSQC spectra. From top to bottom: the *apo* state, the semi*-holo* SL2-RsmE dimer complex (purple) and the semi*-holo* 4bpSL2-RsmE dimer complex (green).

Analysing other regions of the complex in the MD trajectories we saw that the phosphate backbone of the stem of the SL2 RNA formed transient interactions with residues from the β3–β4 loop of both monomers (Fig. 3a and Supplementary Fig. 6a). We observed repeated close approaches (<5 Å) between the E-Q28 Cα in the empty site β3-β4 loop and Cyt20 P in the SL2-stem (Supplementary Fig. 6b). These interactions are consistent with the experimentally observed NH-CSPs in the β3–β4 loops of RsmE dimer between the semi-*holo* and the *holo* states (Supplementary Fig. 7a, b) as well as the CSPs in the side-chain amide of Q28 in the empty β3–β4 loop of the *semi-holo* state (Supplementary Fig. 7c). These interactions could be mediated by sodium ion bridges as suggested by high sodium occupancy at this contact throughout the MD simulations (Supplementary Note 4 and Supplementary Fig. 6a). We therefore investigated the role of electrostatic interactions in the SL2 RNA binding to RsmE dimer by performing SL2-RsmE dimer titrations using ITC with different NaCl concentrations in the buffer solution (Supplementary Fig. 8). Increased ionic strength reduced the first binding affinity proportionally more than the second one supporting the finding that sodium ions play a role in creating a difference between the two binding events.

To investigate the role of the RNA stem contacts with the protein in the anti-cooperativity, a series of experiments were performed using a truncated SL2 RNA with the stem region shortened to 4 base-pairs (4bpSL2 RNA), which modelling studies indicated would be unable to contact the β3–β4 loop of the RsmE empty site. The ITC of the 4bpSL2 RNA binding to the RsmE dimer showed that the affinity is substantially reduced for the first binding event with a remarkable reduction of the enthalpic (−30.15%) and entropic (−34.74%) components as compared to the second binding event (−12.93% and −16.85%, respectively) (Fig. 3b, c). Strikingly, the negative cooperativity observed for the cognate SL2 with a ratio of *K*_D2_/*K*_D1_ of ~20 is reduced to only ~3. Moreover, the semi-*holo* 4bpSL2-RsmE dimer complex showed slightly reduced NH-CSPs as compared to the NH-CSPs in the semi-*holo* SL2-RsmE dimer complex, including the backbone and side chain amides in the empty site’s β3−β4 loop (Fig. 3d and Supplementary Fig. 9, respectively). Interestingly, the semi-*holo* 4bpSL2-RsmE dimer complex lacked the multiple conformational states that were observed in the semi-*holo* SL2-RsmE dimer complex (Fig. 3e, f). These findings indicated that the MD-observed transient contacts between the SL2 RNA stem and the RsmE dimer may increase the binding affinity of the first binding event and showed that the additional semi-*holo* states were dependent on the presence of an extended RNA stem.

In summary, the gain in overall free energy of SL2 RNA binding relative to 4bpSL2 may come from an entropic gain provided by the multiple conformations involving Ade26 in the semi*-holo* complex, as well as enthalpic gains due to an interaction of the RNA stem with the β3–β4 loops of both monomers. Yet the reduced binding affinity persisted for the second 4bpSL2 RNA binding event, with about a 3-fold reduction in affinity on shortening the stem of SL2. (Fig. 3b). This prompted us to further explore the structural and dynamics changes at the empty binding site.

### SL2 RNA binding increases dynamics at the empty binding site

**Fig. 4:**
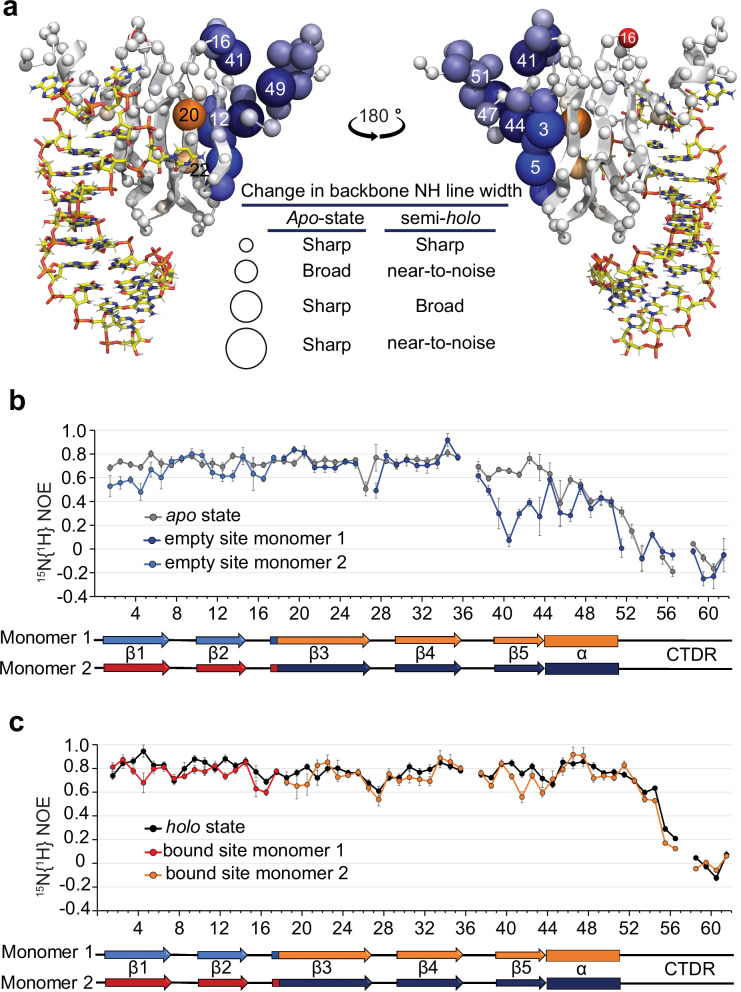
SL2 RNA binding to RsmE dimer enhances motion in its empty binding site. **a** Qualitative mapping of line-broadening in backbone amide peaks in the semi-*holo* SL2-RsmE dimer complex. Spheres highlight the line-broadening of backbone amide peaks. Residues are coloured orange and red in the bound site, and light and dark blue in the empty site. Intensity of colours and the diameter of spheres correlate with the degree of backbone amide peak line-broadening for indicated RsmE residues upon SL2 RNA binding. **b** Plot of ^15^N{^1^H}-heteronuclear NOEs from the *apo* RsmE dimer (grey) and the empty site of the semi-*holo* SL2-RsmE dimer as determined at 303 K. **c** ^15^N{^1^H}-heteronuclear NOE of the *holo* SL2-RsmE dimer complex (black) and the bound site of semi-*holo* SL2-RsmE dimer complex as determined at 303 K. ^15^N{^1^H}-heteronuclear NOE values for both (**b**, **c**) were derived from the ratio of peak intensity in an experiment including ^1^H saturation to one without saturation. Error bars are derived from the standard deviation of the noise in this single pair of spectra.

Three observations for the semi-*holo* SL2-RsmE complex revealed increased dynamics in its empty binding site compared to the *apo* state. First additional line-broadening of backbone amide peaks indicative of motions on the μs–ms timescale, was observed in the ^1^H^15^N-HSQC spectrum for residues in the β1, β2 and β5 strands and the α-helix of the empty binding site as well as for some residues in the β3 strand which links the two binding sites (Fig. 4a and Supplementary Fig. 10). Second the quantitative analysis of Cα, Cβ and C′ chemical shifts indicated that some residues in the empty site α-helix were even less ordered in the semi-*holo* complex than in the *apo* state suggesting a shift in its folded-unfolded equilibrium. (Supplementary Note 5 and Supplementary Fig. 11). Consistent with this partial unfolding, the empty site α-helix also appeared to lose contacts with the β-sheet of the RsmE dimer (Supplementary Fig. 12). Third ^15^N{^1^H}-heteronuclear NOE^9^ experiments showed that binding of one SL2 RNA led to increased sub-nanosecond timescale dynamics of the β1, β2 and especially the β5 strand and the α-helix of the empty binding site as compared to the *apo* state (Fig.4b) whereas much of these additional dynamics were quenched upon binding of the second SL2 RNA (Fig. 4c). Consistent with this, we observed enhanced dynamics (pairwise RMSDs for N atoms) for β5 and the α-helix in the empty site in MD simulations of the semi-*holo* complex when compared to *apo* or *holo* states (Supplementary Fig. 13a). In addition, H-bond partner distances increased and showed greater fluctuation in the semi-*holo* state for the β2–β5 and β5–α helix interactions in the empty site (Supplementary Fig. 13b). We did not observe changes in dynamics for the remaining β-sheet interactions, which might have indicated how SL2-binding changes dynamics of the empty site possibly due to limited timescale of our MD simulations (Supplementary Note 6).

We wondered if the central β-strands of the dimer serve to couple the two binding sites allosterically. That is, the binding of one SL2 RNA may cause structural rearrangements which disrupt the rigidity of the empty binding site and contribute to destabilising the empty site α-helix. To test this hypothesis, the SL2-induced changes in the β-sheets of the RsmE dimer were investigated. Considering that the backbone amides serve as donors in the extensive network of hydrogen bonds (H-bonds) that stabilises these β-sheets, we analysed their temperature dependence to provide information about their strength. The NH-CS are known to shift upfield (lower chemical shifts) linearly with increasing temperatures, resulting in a negative slope which defines the so-called temperature coefficient (Tc)^10^. For hydrogen-bonded amides, subtle chemical shift changes arise from thermal expansion of the H-bond length, leading to weakly negative or even flat slopes. Conversely, the non-hydrogen-bonded amides show steeper negative slopes due to their higher solvent exposure and faster chemical exchange rate^11^. Furthermore, non-linearity in ^1^H-temperature dependence may indicate the presence of alternative conformations. Non-linearity was indeed observed for a third of the backbone amide protons in the *apo* RsmE dimer (Supplementary Fig. 14a). This implies that the RsmE dimer can undergo a temperature-dependent conformational change, with a transition between 303 and 308 K.

**Fig. 5:**
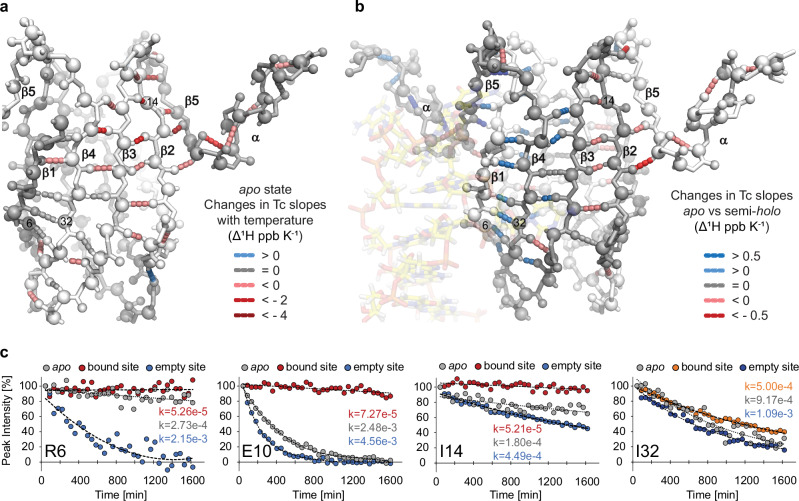
A temperature-dependent structural transition bridges structural and thermodynamic changes between the two binding sites in the RsmE dimer. **a** *Apo* RsmE dimer. Monomers are differently coloured in light and dark grey. Residue numbers label backbone N atoms. Segmented coloured lines indicate H-bonds whose amide experienced non-linear temperature coefficients. (colour-coding indicated in legend). **b** *S*emi-*holo* SL2-RsmE dimer complex. SL2 RNA is rendered transparent to ease RsmE visualisation. Segmented coloured lines indicate H-bonds that changed their amide temperature coefficients upon SL2 RNA binding. **c** HDX curves of R6, E10, I14 and I32 amides in *apo* and semi-*holo* RsmE dimer states, colour-coded using RsmE monomer schemes in Figs. 1, 3 and 4. HDX curves for other backbone amides are shown in Supplementary Fig. 15.

To estimate the magnitude of these structural changes, we calculated the difference between the Tc slopes before and after the transition for every backbone amide (Supplementary Fig. 14b) and mapped the amide protons that underwent the temperature-dependent transition onto the known structure of the RsmE dimer (Fig. 5a and Supplementary Fig. 14c). They clustered in the β2, β3 and β5 strands and the α-helix of the *apo* RsmE dimer. The largest changes corresponded to the hydrogen-bonded amide protons that stabilise the intra-monomeric β2–β3 and inter-monomeric β2–β5 interactions. This analysis of the *apo* RsmE dimer revealed that the β-sheets and the α-helices can undergo coordinated structural changes and access additional states in a temperature-dependent manner.

We next analysed the semi-*holo* SL2-RsmE dimer complex, which also showed multiple residues with a temperature-dependent transition. To assess whether the binding of one SL2 RNA tightened or loosened the H-bonds in the β-sheets of the RsmE dimer, the changes of the Tc slopes between the *apo* and semi-*holo* states were measured (Supplementary Fig. 15a, b). Mapping of these changes onto the known structure of the RsmE dimer revealed two clusters of SL2-induced Tc changes (Fig. 5b and Supplementary Fig. 15c). One cluster involved the tightening of H-bonds that stabilise the β1–β4 β3–β4 β2–β5 interactions and the α-helix in the bound site, as well as some H-bonds that connect the bound β2 strand with β3 in the empty site. In a second cluster, we observed, on the contrary, a weakening of the H-bonds in the β1–β4 and β2–β5 interactions and in the α-helix of the empty binding site, as well as H-bonds connecting the empty β2 strand with β3 in the bound site. These observations are further supported independently by hydrogen-deuterium exchange (HDX) rate changes of backbone amides in the *apo* and semi-*holo* RsmE dimer (Fig. 5c and Supplementary Fig. 16). The binding of one SL2 RNA reduced the HDX rate in several backbone amides in the bound site while increasing HDX rates in the empty binding site. These two independent methods strongly suggest that the binding of one SL2 RNA triggers a concerted structural and dynamic change in the RsmE dimer whereby the dynamics of the bound binding site is quenched, resulting in increasing dynamics at the empty binding site, leading to a partial unfolding of the α-helix at the empty site. The backbone hydrogen-bonding network of two anti-parallel beta-sheets serves as a conduit to connect the two binding sites. The higher conformational entropy associated with increased dynamics at the empty site of the semi-*holo* complex can compensate for the reduced entropy of the binding site, thereby favourably contributing to the free energy of binding. In turn, a second RNA binding in the yet empty site is therefore weakened since a larger entropy change is needed to achieve RNA binding, furnishing an explanation for the observed anti-cooperativity.

## Discussion

The investigation of anti-cooperative binding in biological systems profits from the relative ease of populating and characterising the intermediate states. From a static perspective, some crystallographic studies have shown that binding of the first ligand can deform the distant empty site, with the expected consequence of an unfavourable binding for a second ligand^12^. Conversely, other crystallographic studies on intermediate states showed no obvious structural changes on the unliganded sites that could explain the negative cooperativity^13^. NMR studies in solution have shown that negative cooperativity can indeed occur with no conformational changes bridging the two binding sites^14^. Our investigation on the intermediate states of RsmE dimer provided a mixed picture. We could explain why the binding affinity at the first binding site is stronger than at the second binding site. For the semi-*holo* state, we found multiple lines of evidence indicating that RsmE accesses more conformations in both the RNA bound site and especially at the empty site compared to the *holo* forms (Figs. 2 and 4). This suggests that the semi-*holo* state has higher conformational entropy compared to the *holo* state, which could contribute to the more favourable binding free energy for the first SL2 binding. In addition, the first RNA binding makes additional interactions via the stem with the β3-β4 loops in the RsmE dimer (Fig. 3a), which contribute to binding enthalpy while also contributing to the large entropic penalty incurred in the canonical RNA binding site (α-helix β1 and β5 strands). Some of this entropic penalty is offset by increased dynamics elsewhere, which is allosterically communicated to the empty site, contributing favourably to the first binding event via increased entropy. Conversely, these increased dynamics in the accessible binding site augment the entropic penalty for the second RNA binding event. The second binding event also causes subtle structural changes in the initially bound site via allostery (Fig. 1b and Supplementary Fig. 7), reducing the overall enthalpy due to less favourable interactions in the initially bound site. Altogether, from a thermodynamic point of view, our results explain well the origin of the observed difference in RNA binding affinity between the first and second binding events.

The second and unexpected aspect of our work relates to the allosteric pathway that couples the two binding sites. We observed no major changes in the protein dimer upon RNA binding to the first binding site with only modest chemical shift differences between semi-*holo* and *holo* states (Fig. 1c). However, close examination of the hydrogen-bond strength in the β-sheets of the RsmE dimer revealed a rigidification of the β-strands at the RNA-bound site and a relaxation or weakening in the β-strands of the empty site (Fig. 5b, c). This supports a model in which the backbone of the β-strands communicates an allosteric signal from the bound to the distant empty site without major structural changes in the transition region. In the past, we^15,16^ and others^17,18^ observed that RNA binding triggered rigidification at the binding site with increased dynamics in other parts of the molecule. This can be interpreted as a conservation of conformational entropy during the binding process: the entropy loss at the RNA binding surface of the protein is compensated by increased conformational entropy at a distant site. This is what we clearly observed here as well, except that this conformational entropy compensation is now also functionally important in multivalent binding as it results in a lower binding affinity for the second RNA molecule.

Importantly, increasing the temperature—and thereby entropic effects—of the *apo* RsmE dimer in isolation led to similar relaxation of H-bonds in both binding sites (Fig. 5a) in the absence of RNA binding. This observation suggests that the fold of the *apo* binding sites is inherently susceptible to subtle changes in conformational entropy. Thus, the two binding sites can be considered as two energetically frustrated structural elements^19^ where the cognate RNA binding eliminates local frustration, but enhances energetic frustration on the other binding site.

**Fig. 6:**
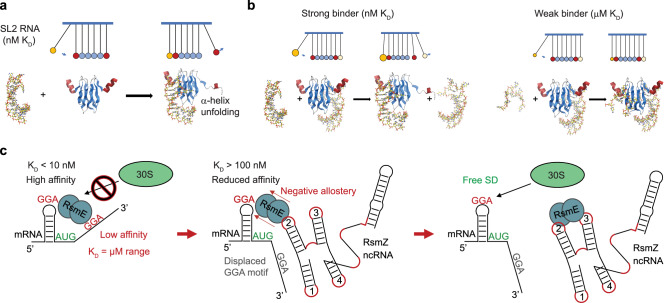
Newton’s cradle, the negative allostery in the RsmE dimer and the de-repression of mRNA translation in bacteria. **a** Newton’s cradle analogy for the binding of SL2 RNA to RsmE dimer and the emergence of a high-energy state on the empty binding site. **b** Newton’s cradle analogy for the understanding of the affinity-associated allostery. **c** The biological function of the negative allostery in the RsmE dimer.

In the RsmE dimer, we observe how conformational entropy apparently propagates through a network of β-strands connecting the two RNA-binding sites, creating a dynamic coupling analogous to Newton’s cradle (Fig. 6a). Entropy-driven allosteric mechanisms^20^ have been observed in various systems, including Newton’s cradle-like dynamics in bimetallic porphyrin complexes^21^. Similarly, entropy redistribution enables allosteric communication in metalloregulatory proteins akin to the energy propagation in Newton’s cradle^22^. In T4 lysozyme, cooperative allosteric transitions rely on concerted motions within a flexible transmission network^23^, whereas in the murine urokinase-type plasminogen activator, a disordered light chain enhances protease activity by transmitting allosteric effects through β-strands, altering distant loop dynamics and catalytic activity similar to Newton’s cradle-like propagation^24^. In a similar manner, allostery is mediated across domains by subtle rearrangements of conformational sampling in β-sheets of the PPIase^25^ and WW domains^26^ in Pin1 and PDZ domains^27^. McLeish et al. proposed that fluctuation-driven allostery can be conceptualised through emergent collective motions^28^. They show how the coupling of these collective motions between the two homotypic monomers of catabolite activator can lead to the observed strong negative allostery of cAMP binding. They predict enhanced dynamics at the non-bound site, which is quenched by the second binding event, consistent with the behaviour of the RsmE dimer. Kornev^29^ proposed that entropy-driven allosteric communication arises from the emergent behaviour of self-organised oscillators, forming semi-rigid clusters that transmit signals efficiently without necessitating structural rearrangements. Our findings suggest that the RNA-binding mechanism of the RsmE dimer leverages a similar entropy-driven process to achieve functional coordination, where the β-strand network acts as a rigid conduit for transmitting conformational entropy, enabling functional communication across distant sites.

In the Newton’s cradle analogy, the binding RNA stem-loop can be seen as the incoming ball, while the β-strands of the RsmE dimer represent the middle balls. In an ideal Newton’s cradle, the kinetic energy imparted to the outer ball is transferred through the middle balls without apparent displacement and ultimately to the ball on the opposite side, which then moves away but can rebind. By analogy in our system, the kinetic energy may be replaced by conformational entropy that is transmitted via the β-strands, leading to unfolding of the helix on the other side (Fig. 6a). The analogy with the cradle also comes from the in-line compression and expansion that is transmitted by the hydrogen-bond network. The cradle analogy furnishes a rationale for why we cannot detect anticooperativity with weak RNA binding^7,8^: weak binding results in insufficient conformational entropy transfer to destabilise the distant binding site, and therefore fails to weaken the affinity for a second RNA (Fig. 6b). Similarly, if a second RNA binds to the second site with very high affinity, the process can reverse causing increased entropy at the initially bound site, which can lead to lower affinity and partial dissociation of the initially bound RNA on the other side. This lowered affinity at the second binding site is functionally important as it allows us now to explain how the RsmZ ncRNA can displace tightly bound RsmE dimers from the bound mRNAs. In light of our results, we can envisage that this happens in two steps (Fig. 6c). In a first step, SL2 of the ncRNA RsmZ displaces the weakly bound mRNA because of the high affinity of SL2 for binding RsmE. SL2 binding then allosterically triggers increased motion in the second RNA binding site that is bound by the SD stem-loop and therefore entropically destabilises it. This destabilization is consistent with the ~30-fold reduction in SD RNA affinity when the other site is bound by SL2 (Supplementary Fig. 1a, b and Supplementary Table 1). RsmZ SL3, which is in cis with SL2, can then compete with and displace the SD sequence, resulting in the detachment of the RsmE dimers from the mRNAs and leading to mRNA de-repression and ultimately gene expression. We anticipate that similar mechanisms may facilitate the displacement of multiple RNAs in other multivalent RNA-binding proteins.

In general terms, the negative cooperativity observed in the RsmE dimer illustrates mechanistically how evolution can fine-tune protein folds and frustrated binding sites to thermodynamically couple distant allosteric and functional sites. This thermodynamic coupling can regulate in an energetically efficient manner diverse biological process including cAMP sensing and the associated gene expression in bacteria^14,30,31^, calmodulin function^32^, cell growth and proliferation via PDZ domains in mammals^33^ biochemical processes such as enzyme catalysis in monomeric proteins^34^ communication in homodimeric and multidomain proteins^35,36^ and even pathological processes such as the toxicity of prion proteins^37^.

## Method

### Expression and purification of the RsmE dimer

The coding sequence of RsmE dimer was cloned in the pET28b plasmid (Novagen), which added a 6X-His tag to the C-terminal end of RsmE. To ease the quantification of the RsmE dimer molar concentration a Proline-to-Tryptophan (P64W) mutation was introduced at the C-terminal end of RsmE to increase the 280 nm UV absorbance of the RsmE dimer. Plasmid DNA sequence was verified using Sanger sequencing, and BL21-Codon Plus (DE3)-RIL *E. coli* cells (Agilent Technologies) were transformed with pET28b-64WRsmE for protein expression. Once the bacterial culture reached an OD_600_ of 0.6–0.8, expression was induced with 1 mM isopropyl-β-d-thiogalactopyranoside (IPTG) and carried out for 4 h at 37 °C. For isotopic labelling of RsmE dimer, *E. coli* cells were grown in M9-minimal medium containing 99%−^15^NH_4_Cl and either D-glucose or 99%−^13^C_6_-D-glucose to express either uniformly ^15^N-labelled RsmE dimer or ^13^C^15^N-labelled RsmE dimer. For the perdeuterated RsmE dimer sample, the M9 medium was prepared in 100% D_2_O, instead of H_2_O. *E. coli* cells were pelleted by centrifugation and resuspended in lysis buffer: 50 mM potassium phosphate, pH 8, 300 mM NaCl, 10 mM imidazole and protease inhibitor cocktail (cOmplete EDTA-free, Roche). The cells were lysed using a microfluidizer (Microfluidics), and the clarified lysates containing RsmE dimer were loaded on Ni-NTA-agarose beads (Qiagen). The Ni-bound RsmE dimers were washed with a solution containing 50 mM potassium phosphate 1 M NaCl 10 mM imidazole pH 8 followed by stepwise washes with increasing concentration of imidazole (30 and 100 mM) and RsmE dimers were eluted with 250 mM imidazole. Proteins were dialysed overnight against NMR buffer (30 mM NaCl, 50 mM potassium phosphate, pH 7.2) and concentrated with a centricon (3 kDa molecular mass cut-off membrane Vivascience) to reach the desired protein concentration. Samples were tested for purity by SDS-PAGE (Laemmli, 1970). Protein concentrations were determined by their 280 nm UV absorption, and the theoretical Molar extinction coefficient of the RsmE dimer (6990 M^−1^ cm^−1^) was calculated using the ExPASy tool ProtParam^38^.

### RNA in vitro transcription and purification

SL2 RNA (5′-GGGCCAUCAAGGACGAUGGUCC-3′), hcnA SD RNA (5′-GGGCUUCACGGAUGAAGCCC-3′) and 4bpSL2 RNA (5′-GGAUCAAGGACGAUCC-3′) were in vitro transcribed from double-stranded DNA templates (Microsynth) using in-house-produced T7 RNA polymerase and unlabelled triphosphate nucleotides for transcription^39^. The products were purified by anion exchange chromatography under denaturing conditions, followed by butanol extraction as previously described^40^. Pelleted RNAs were resuspended in water, incubated at 98 °C for 1 min and slowly cooled for refolding. RNA purity was tested using urea-PAGE. Concentrations were determined using the 260 nm UV absorbance. Their theoretical molar extinction coefficient (SL2: 243.14 mM^−1^ cm^−1^ hcnA SD = 216.04 mM^−1^ cm^−1^ and 4bpSL2 = 183.1 mM^−1^ cm^−1^) and final concentration were calculated using the online tool RNACalc^41^.

### NMR spectroscopy and NMR data analysis

NMR experiments were recorded at 303K and 313 K for the apo ^15^N or ^13^C^15^N-RsmE dimers and all other RNA-RsmE dimer complexes on Bruker Avance III 500, 600, 700 or 900 MHz spectrometers. All shown spectra and associated NMR data were measured at 313K unless indicated otherwise. All spectrometers were equipped with cryoprobes and running Topspin 3. All samples were prepared in NMR buffer (30 mM NaCl, 50 mM potassium phosphate, pH 7.2) with 3% D_2_O. Protein concentration was either 300 or 600 µM. For protein assignment experiments of the semi-holo (1:1) complex a slight excess of SL RNA was used (1.1:1). For NOESY spectra and ^1^H^15^N-HSQC spectra used to assess line-broadening the ratio was 1:1 and a protein concentration of 600 µM was used. Resonance assignments were based upon the previously obtained assignments of the *holo* RsmE dimer-SL2 complex (BMRB accession code 19546). Complete assignments of the ^1^H^15^N-HSQC spectra for the *apo* RsmE dimer and SL2 RNA-bound RsmE dimer were obtained with triple resonance spectra (HNCACB, HNCA, HN(CA)CO and HNCO)^42^. To assign ^1^H^13^C chemical shifts of the amino acid side chains, 3D HCcH Total correlation spectroscopy (TOCSY) and 3D HCH-Nuclear Overhauser effect spectroscopy (NOESY) experiments were recorded. NOE mixing times of 120 ms were used except for filtered edited NOESY experiments, which used 150 ms. RsmE-bound SL2 RNA chemical shifts were retrieved from the PDB entry 2MFE of the solution structure of RsmE dimer bound to two SL2 RNAs. For ^1^H^13^C-HSQC 3D HCcH-TOCSY 3D HCH-NOESY and filtered-edited NOESY experiments, protein or RNA-protein samples were lyophilised overnight and resuspended under low humidity conditions in 100% D_2_O. For ^15^N{^1^H}-heteronuclear NOE experiments, perdeuterated ^15^N-RsmE dimer samples were prepared, and spectra were recorded at 303 K. ^1^H irradiation was applied for the last 3 s of a 5 s recycle delay and was omitted in the reference experiment. Data were analysed with DynamicsCenter 2.5, and errors were estimated from the signal-to-noise of individuals peaks. NMR data were processed with Topspin 3.0 software. NMR spectra were analysed in NMRFAM-SPARKY software^43^. Backbone amide chemical shift perturbations (Δδ_NH_) were calculated with the following equation:

1 $${{{\Delta \delta }}}_{{{\mathrm{NH}}}}=\sqrt{{{{{\Delta \delta }}}_{{{{\rm{H}}}}}}^{2}+{\left({0.153}{{{\Delta \delta }}}_{{{{\rm{N}}}}}\right)}^{2}}$$

where Δδ_H_ and Δδ_N_ are the ^1^H and ^15^N chemical shift difference between the compared states.

For the calculation of random coil chemical shifts of the *apo* RsmE amino acid sequence at 313 K and pH 7.2, the chemical shifts and the sequence correction factors were derived as reported by Kjaergaard and Poulsen^44^. The basis to determine the sequence correction factors was described in Schwarzinger et al.^45^. The glycine correction factors and the temperature coefficients were derived from Kjaergaard et al.^46^.

For the analysis of the NH peak line shape, summarised in Fig. 4a, a qualitative approach was applied. Although a quantitative line shape analysis was attempted, it was not possible to compare the semi-*holo* empty site to the *apo* state due to the poor signal/noise (S/N) ratio and/or extensive line-broadening in multiple peaks from the empty binding site of the semi-*holo* complex. To perform a qualitative comparison, each peak was classified using two values: its S/N ratio and its line shape. When a peak’s S/N ratio exceeded the average peak S/N ratio in the spectrum by at least one standard deviation, and the ^1^H line-shape could be fit to a Lorentzian function in SPARKY, the peak was classified as 'sharp'. When a peak fulfilled only one of the two criteria, the peak was classified as 'broad', and when a peak failed to fulfil either criterion, it was classified as 'near-to-noise'. After classification, the changes in peak classification from the *apo* state to the semi-*holo* state were determined, and they were represented as sphere of different sizes in the structural model in Fig. 4a, following the scale that is provided in the centre of the figure.

### Determination of amide proton temperature dependence

^1^H^15^N-HSQC experiments from *apo* RsmE dimer and SL2-bound RsmE dimer were acquired at 283, 288, 293, 298, 303, 308, 313, 318, 323 and 333 K. After heating to 333 K, thermal reversibility was checked by returning to 298 K. The *apo* RsmE dimer and SL2 RNA-bound RsmE dimer spectra were completely reversible. A sample of 1 mM sodium 3-(trimethylsilyl)propane-1-sulfonate (DSS) in NMR buffer (30 mM NaCl, 50 mM potassium phosphate, pH 7.2) with 3% D_2_O was used to reference the ^1^H chemical shifts at different temperatures. Chemical shift changes were monitored as a function of temperature. Temperature coefficients and their error were obtained from linear regression fit of the slope in ppb K^−1^. NMR assignment of ^1^H^15^N-HSQC spectra at different temperatures was conducted by manually tracking the systematic shifts. The assigned ^1^H^15^N-HSQC spectrum at 313 K was used as a reference.

### Hydrogen-deuterium exchange

A series of ^1^H^15^N-HSQC experiments were recorded at 313 K after RsmE dimer samples dissolved in NMR buffer and lyophilised were redissolved in D_2_O. Exchange rates were determined from the time dependence of peak intensities. The decay curves were fitted to a first-order rate with R version 5.4:

2 $$h\left(t\right)=A\,\exp \left(-{kt}\right)$$

where *h(t)* is the NH-CS peak height at time *t* $A$ is fitted to the initial intensity, and *k* is the exchange rate. The best fit for the exchange rate *k* is reported.

### Isothermal titration calorimetry (ITC)

Prior to ITC experiments, purified RsmE dimer and RNA samples were dialysed against the same NMR buffer (buffer 30 mM NaCl, 50 mM potassium phosphate, pH 7.2), and the calorimeter was calibrated following the manufacturer’s instructions. All ITC experiments were performed on a VP-ITC instrument from MicroCal, where RNA was titrated into the RsmE protein. RsmE monomer concentration in the cell (cell volume = 1.4644 mL) was calculated to be 10 μM. The syringe was loaded with 280 μL of the RNA, also in NMR buffer (80–100 μM concentration). Experiments were conducted at 298 K, typically consisting of 40–70 injections of 4–10 µl. The injection speed was 2 s/µl with 5 min interval between injections. The syringe stirred at a rate of 307 rpm. Data were integrated and fitted to a sequential binding model using Origin 7.0 software. This model implicitly assumes two binding events, and so N is not a fitting parameter. The dissociation constants (*K*_d1_ and *K*_d2_) and the enthalpic terms (Δ*H*_1_ and Δ*H*_2_) were derived directly from the fitted model. The entropic terms (−*T*Δ*S*_1_ and *−T*Δ*S*_2_) were calculated using the standard Gibbs’ Free energy equation ($\Delta G=\Delta H-T\Delta S$), where Δ*G* was calculated from the following equation:

3 $$\Delta G=-{{{\rm{RT}}}}\,{{{\mathrm{ln}}}}\,{K}_{{{{\rm{d}}}}}$$

ITC experiments were performed in biological triplicate (*n* = 3) to verify reproducibility and to estimate experimental error. No statistical methods were used to predetermine sample sizes, as *n* = 3 is standard for quantitative biophysical measurements of purified systems. Thermodynamic parameters and individual replicate numbers are listed in Supplementary Table 1. The mean and standard deviation of the *K*_d_ values and thermodynamic parameters were calculated from three ITC replicates.

### Molecular dynamics simulations

Conformer 13 of the NMR solution structure bundle of RsmE dimer bound to two SL2 RNA (PDB: 2MFE) was used as the starting structure in all MD simulations. Zero one or two SL2 RNA were removed prior to solvation (explicit-solvent conditions) and equilibration of *apo*, semi-*holo* and *holo* states, respectively. We have used the xLeap module of AMBER18 to prepare the topology and coordinate files. A summary of the system setup is provided in Supplementary Table 2. Three or four simulations were performed for each system. All simulations ran for the minimal length production of 10 μs. The ff14SB and bsc0χOL3 force fields were implemented to describe protein and RNA potentials, respectively^47,48^. In our experience, this MD simulation system setup often provides insightful observations that can then be further investigated using experimental methods such as NMR spectroscopy. The simulations were performed using AMBER18 software. Standard protocols for equilibration and simulation of protein/RNA complexes were applied^49^. Each system underwent a pre-production equilibration and minimisation. Positional restraints of 25 kcal/mol/Å^2^ were placed on the RNA for the first minimisation. Subsequently, using the same positional restraints, the system was heated in an equilibration run from 100 to 300 K on the timescale of 10 ps. Then a series of six minimisations and equilibration runs were performed with positional restraints on the RNA of 5, 4, 3, 2, 1 and lastly, 0.5 kcal/mol/Å^2^ for 5 ps each. Each minimisation involved 500 steps using the steepest descent method and then 500 steps using the conjugate gradient method. Production simulations were performed with the pmemd.cuda^50^, using periodic boundary conditions and an NPT ensemble at 300 K. All atomistic distances, H-bond distances and pairwise root mean square deviations (pRSMD) were calculated from the trajectories using the cpptraj module in AMBER18. Prior to pRSMD measurements, all frames were aligned using the amino acid backbone atom coordinates of the rigid core of the RsmE dimer (residues 1–44). Information on the analysis of fractional sodium occupancy in MD simulations can be found in Supplementary Note 4.

To obtain the 10 representative structures reported in Fig. 2d, e we extracted all the frames in the MD simulations that matched the two maximum peaks that were observed in the 2D-plot correlating the distances Ade26(H2)–B-I47(Hγ2) and Ade(H2)–B-A57 (Hβ) (Fig. 2d). For bound state Ι we retrieved all frames that showed an Ade26(H2)–B-I47(Hγ2) distance between 4.8 and 4.9 Å and an Ade(H2)–B-A57 distance between 2.9 and 3.0 Å. For bound state ΙΙ we retrieved all frames that showed an Ade26(H2)–B-I47(Hγ2) distance between 5.0 and 5.1 Å and an Ade(H2)–B-A57 (Hβ) distance between 11.0 and 11.1 Å. In both cases, a few thousand frames were retrieved, and 10 representative frames were randomly selected their Cα-backbones were aligned, and they are displayed in Fig. 2e.

### Reporting summary

Further information on research design is available in the Nature Portfolio Reporting Summary linked to this article.

## Supplementary information

Supplementary Information

Reporting Summary

Transparent Peer Review File

## Source data

Source Data

## Data Availability

Code and parameter files for the equilibration and production of MD simulations are available in the Zenodo repository [10.5281/zenodo.18427853].

## Data availability

All data necessary to support the conclusions of this study are provided in the main text, main figures, Supplementary Information, and Source data files. NMR spectra and ITC data files, used for quantification, are available from the corresponding author upon request. MD trajectories used for quantification have been deposited at MDrepo MDR00020893 [https://mdrepo.org/explore/20893], MDR00020894 [https://mdrepo.org/explore/20894], MDR00020895 [https://mdrepo.org/explore/20895], MDR00020896 [https://mdrepo.org/explore/20896], MDR00020897 [https://mdrepo.org/explore/20897], MDR00020898 [https://mdrepo.org/explore/20898], MDR00020899 [https://mdrepo.org/explore/20899], MDR00020900 [https://mdrepo.org/explore/20900]) and starting coordinates for MD runs at Zenodo with accession code 18334758. A source data file for this paper is also available at Zenodo under accession code 19081039. The coordinates for the *holo* dimer of RsmE used to model the semi-*holo* complex are available from the protein databank under accession code 2MFE. The chemical shifts used as a starting point for assigning signals in the semi-*holo* state are available from the Biological Magnetic Resonance Databank under accession code 19546. Chemical shifts obtained for the *apo* state at 303 K and 313 K, and for the semi-*holo* state at 313 K have been deposited at the BMRB databank with accession codes BMRB 53701 (*apo* 303 K), BMRB 53702 (*apo* 313 K), and BMRB 53703 (semi-*holo*), respectively. Source data are provided with this paper.
