# Supplementary material for: Newton’s cradle-like allosteric mechanism explains regulatory RsmE RNA binding
Source: Nat Commun. 2026 Apr 22;17:5545. doi: 10.1038/s41467-026-72126-z (PMC13287468; doi:10.1038/s41467-026-72126-z)
Supplement: Supplementary file 1 — Supplementary Information [file 41467_2026_72126_MOESM1_ESM.pdf]

Supplementary Information File for:

"Newton's cradle-like allosteric mechanism explains regulatory RsmE RNA binding"

Esteban Finol[1], Fred F. Damberger[1], Miroslav Krepl[2], Timo Flügel[1], Priscilla Dietrich[1], Thomas C.T. Michaels[1], Beat Vögeli[3], Jiří Šponer[2], Frédéric H-T. Allain[1,*].

[1] Institute for Biochemistry, Department of Biology, ETH Zurich, 8093, Zurich, Switzerland

[2] Institute of Biophysics of the Czech Academy of Sciences, Kralovopolska 135, 61200, Brno, Czech Republic

[3] Department of Biochemistry and Molecular Genetics, University of Colorado Anschutz Medical Campus, Aurora, CO 80045, USA.

*To whom correspondence should be addressed.
Email address: allain@bc.biol.ethz.ch

Supplementary Figures:

Supplementary Figure 1: The high affinity binding of hcnA SD RNA to RsmE dimer.

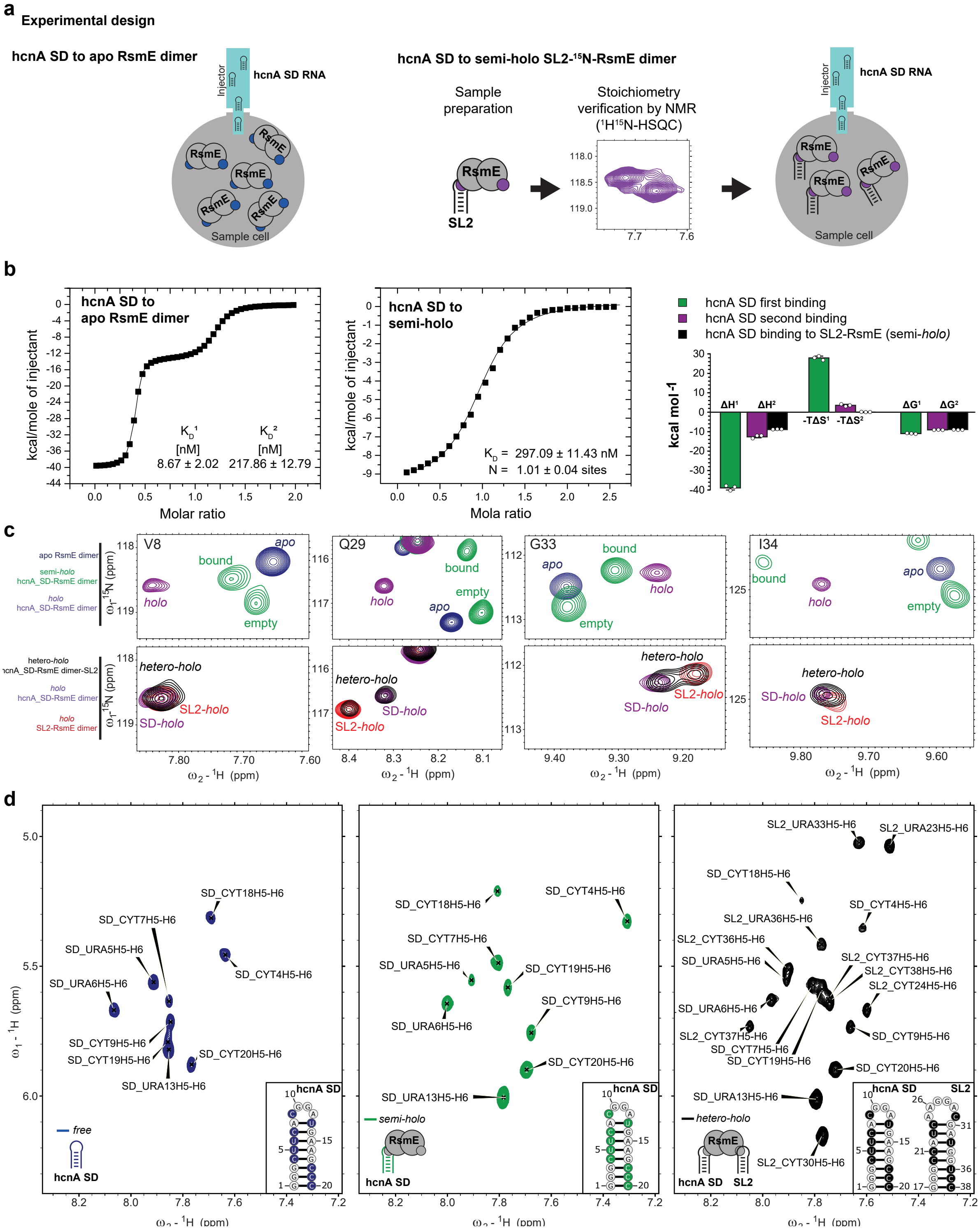

a, Scheme showing the experimental design to determine the effect of SL2 RNA binding on the affinity of RsmE dimer for the hcnA SD RNA using ITC measurements. **b**, ITC-derived binding curves of hcnA SD RNA titrated to the *apo* and semi-*holo* RsmE dimer. The molar ratio corresponds to the RNA:RsmE monomeric ratio. A bar-plot with thermodynamic changes for the first and second binding events. Values can be found in Supplementary Table 1. **c**, isolated backbone amide peaks in the overlaid $^{1}H^{15}N$-HSQC spectra of the *apo* (blue), semi-*holo* (green) and *holo* (purple) hcnA SD-^{15}N-RsmE dimer states. An additional set of $^{1}H^{15}N$-HSQC spectra show the overlay of the hetero-*holo*, SD-*holo* and SL2-*holo* complexes. **d**, Assignment of the $^{1}H^{1}H$-TOCSY of hcnA SD and its semi-*holo* and hetero-*holo* complexes.

Supplementary Figure 2: The semi-*holo* SL2-RsmE dimer complex has two additional bound RsmE states.

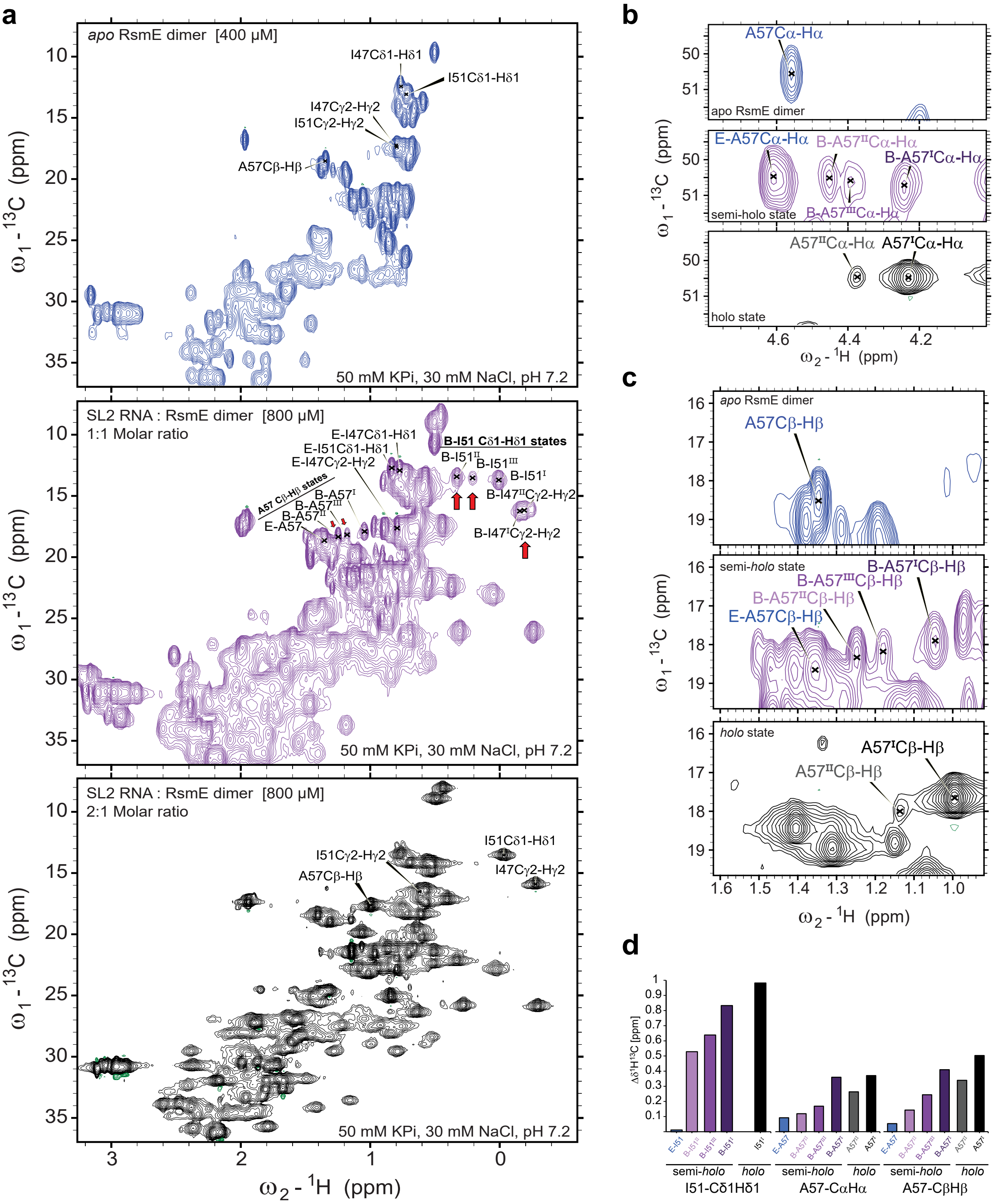

a, Methyl region of $^{1}H^{13}C$-HSQC spectra from the *apo* ^{13}C-labelled RsmE dimer (top), the semi-*holo* (middle in purple) and *holo* (bottom in black) SL2 RNA-bound RsmE dimer. Red arrows indicate the minor conformation peaks in the semi-*holo* complex. **b** & **c**, A57 CαHα and A57 Cβ-Hβ peaks of different states in $^{1}H^{13}C$-HSQC spectra. Top: the *apo* RsmE dimer. Middle: the semi-*holo* SL2-RsmE dimer complex. Bottom: the *holo* SL2-RsmE dimer complex. **d**, Bar plots with the CSPs of two methyl groups in the RsmE dimer for the different conformations upon binding of one SL2 RNA: one methyl is in the α-helix (I51Cδ1-Hδ1) and the other is in the CTDR (A57Cβ-Hβ).

Supplementary Figure 3: Assignment of secondary conformations in the bound α-helix and CTDR of the RsmE dimer.

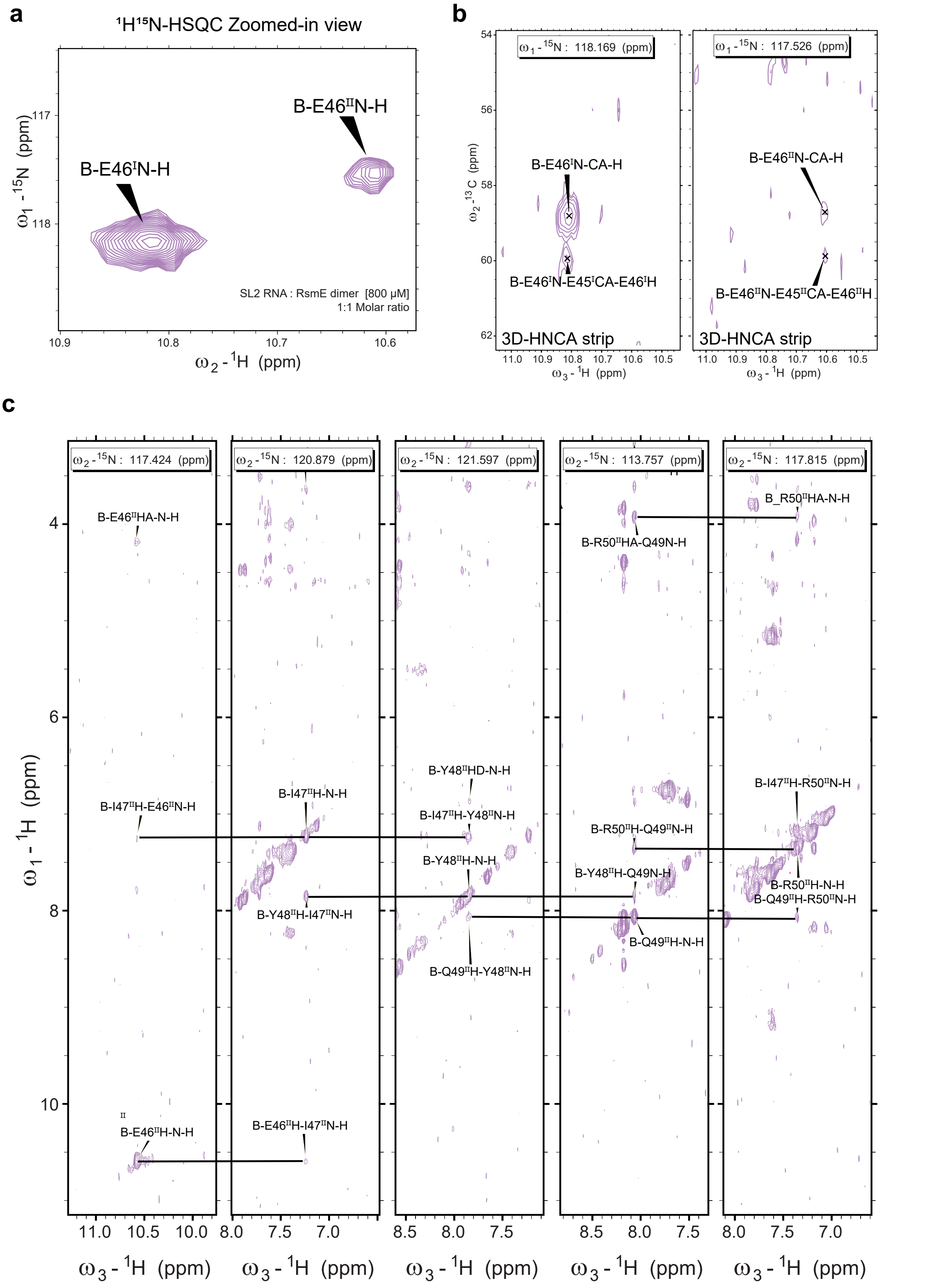

a, Region of $^{1}H^{15}N$-HSQC spectrum with B-E46 NH peaks from the semi-*holo* SL2-RsmE dimer. **b,** Strip plots from HNCA spectrum of semi-*holo* RsmE dimer state. Strips are centred on ^{15}N-CS of major E46^{I} and minor E46^{II} backbone amide peaks. **c,** Strip plots from ^{15}N-resolved NOESY spectrum of RsmE dimer bound to one SL2 RNA. Strips are centred on the NH-CS of residues 46^{II} to 50^{II} from minor conformation of the semi-*holo* SL2-RsmE dimer complex. Black lines indicate sequential assignment.

Supplementary Figure 4: State II conformation is stable in μs timescale MD.

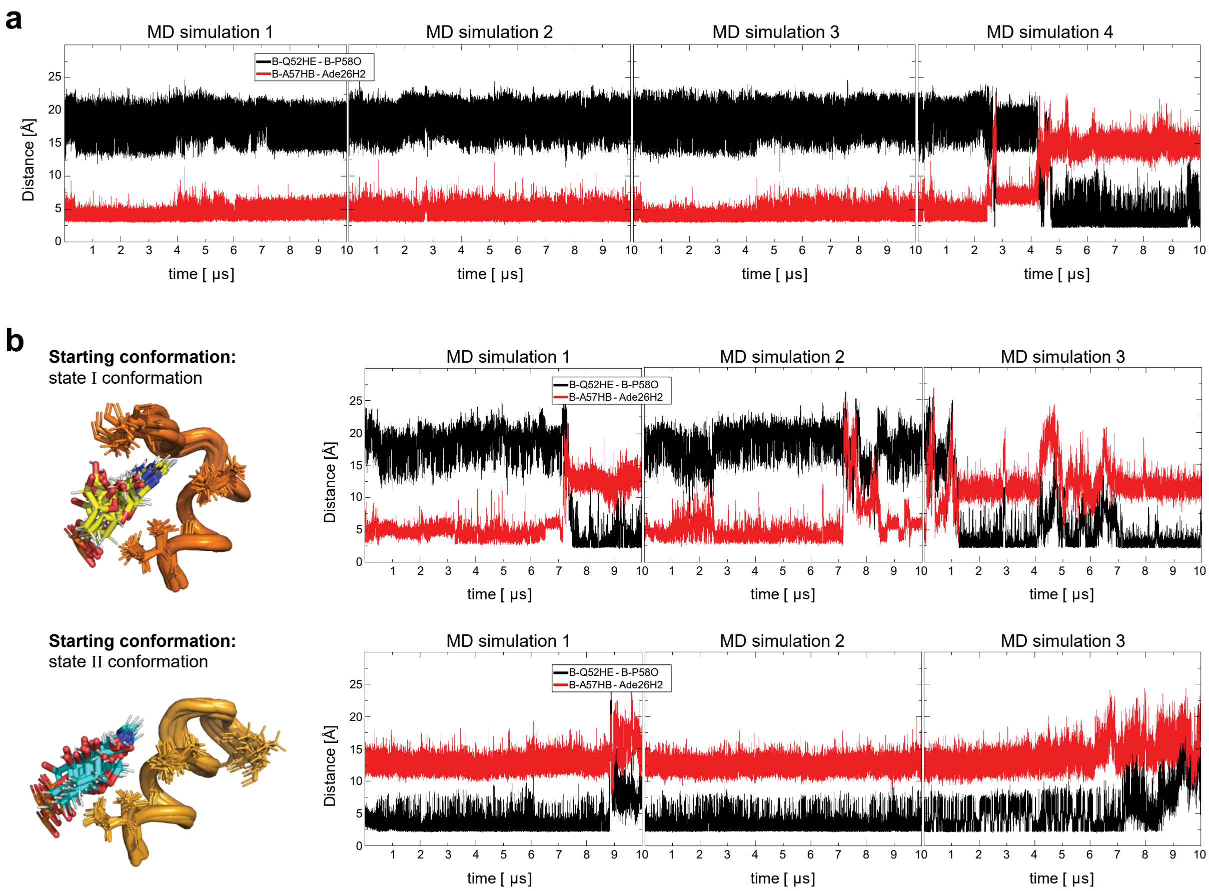

a, Evolution of atomistic distances B-A57Qβ-Ade26H2 (red) and B-Q52Hε-P58O (black) in four MD simulations from the semi-*holo* SL2 RNA-RsmE dimer complex. **b**, Evolution of the same atomistic distances in MD simulations that started from Ade26-bound state I (top panel) and state II (bottom panel) conformations. Starting conformations of the CTDR where Adenine 26 interacts as in the SL2 RNA-RsmE dimer complex (top), and the conformation where the CTDR loses the contact to Ade26 (bottom). The measured distances are indicated by red and black lines.

Supplementary Figure 5: Minor conformation II corresponds to the MD-derived state conformation in the CTDR of the bound RsmE dimer.

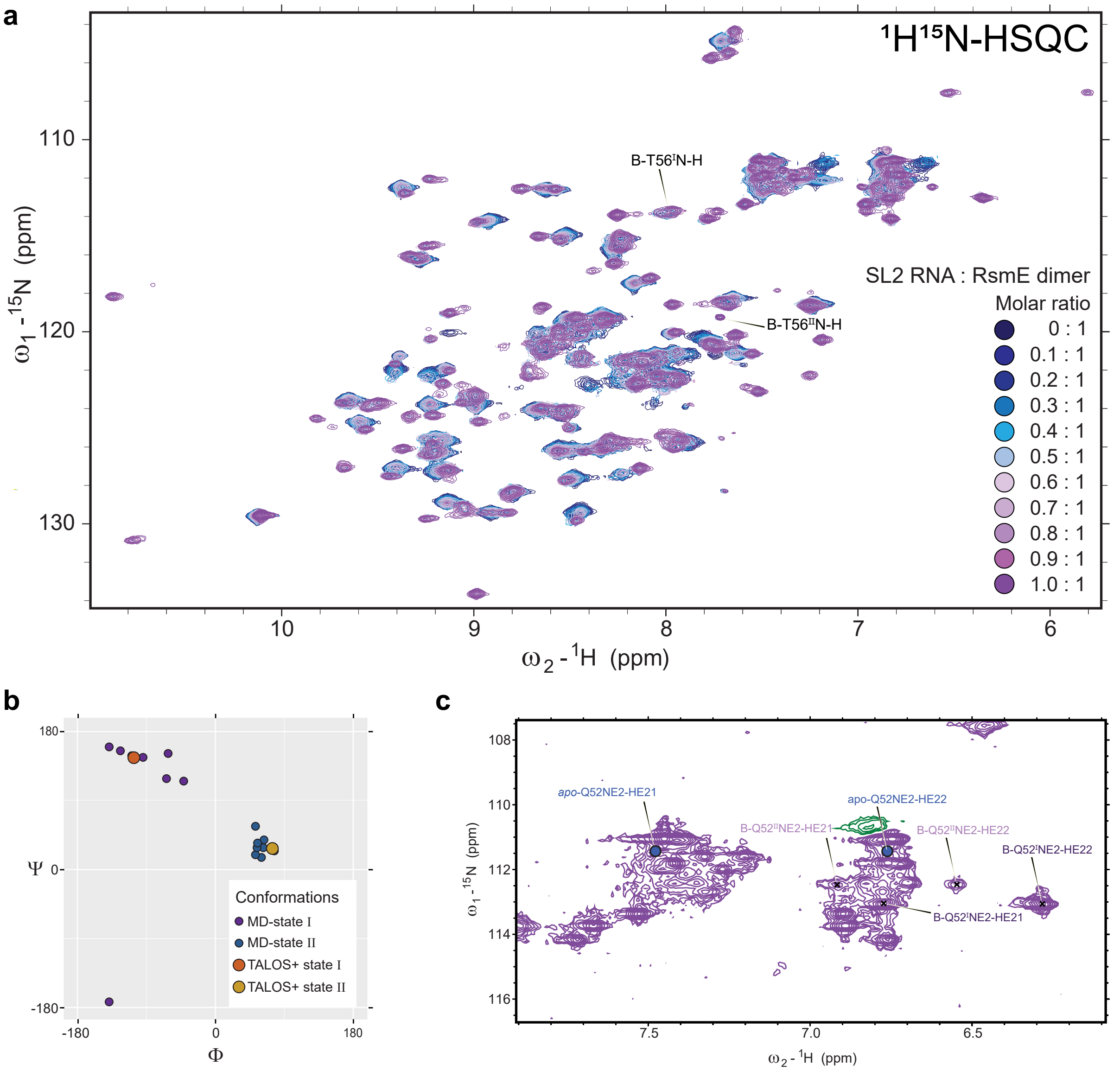

a, Overlaid ^{1}H^{15}N-HSQC spectra from the titration of SL2 RNA into ^{15}N-RsmE dimer. In this titration, only the first binding site was saturated. As SL2 RNA concentration increased, a set of weak additional peaks emerged. Labels show the B-T56 NH-CS for the major (T56^{I}) and minor (T56^{II}) conformations. **b**, Ramachandran plot with ψ and ϕ angles for B-T56 major (state I) and minor (state II) conformations as predicted from their backbone chemical shifts (H, N, CO, CA) using TALOS+[4]. The ψ and ϕ angles were also calculated from 10 frames of MD state II missing the CTDR-Ade26 contact, and 10 frames from the canonical Ade26-bound MD state I conformation. **c**, Side chain region of the ^{1}H^{15}N-HSQC spectrum of the semi-*holo* RsmE dimer state. Distinct B-Q52 NE2-HE21 and NE2-HE22 CSs were observed for the minor conformation (state II).

Supplementary Figure 6: Transient contacts between the SL2 RNA stem and the β3-β4 loop in the empty site of the RsmE dimer observed in MD simulations.

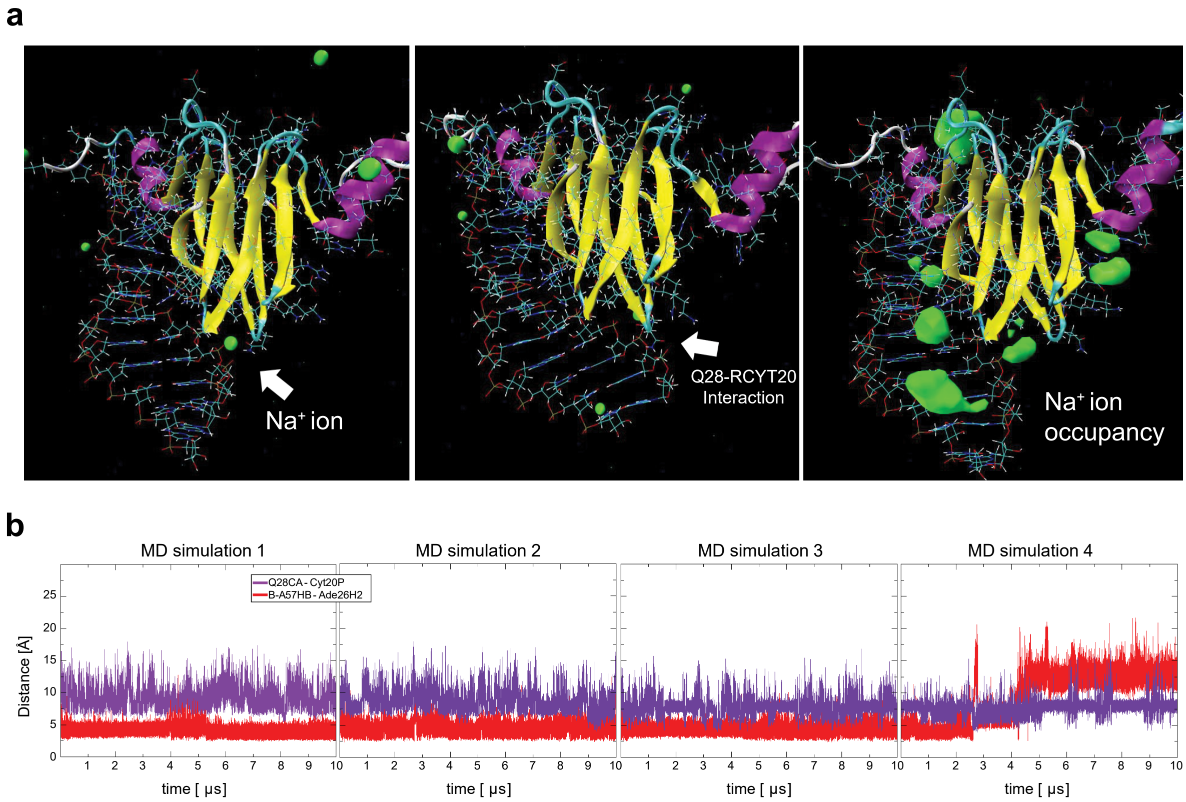

a, Snapshots from the MD simulation of the semi-*holo* SL2-RsmE dimer complex. Secondary structures of the RsmE dimer are shown with yellow (β-strand) and magenta (α-helix) ribbons. Sodium ions (Na^+) are shown as green spheres in the first two panels. The white arrows point to the "coordinated" sodium ion in the first panel, and to the contacts between the RNA stem and the β3-β4 loop of the empty site of RsmE dimer in the second panel. Third panel shows the sodium ion occupancy with green volumes indicating regions of higher occupancy (>5%) throughout the MD simulations. **b**, Evolution of E-Q28Cα-Cyt20P (purple) and B-A57Qβ-Ade26H2 (red) atomistic distances in four MD simulations of the semi-*holo* SL2-RsmE dimer complex.

Supplementary Figure 7: Evidence for transient contacts between SL2 RNA stem and β3-β4 loops in RsmE dimer.

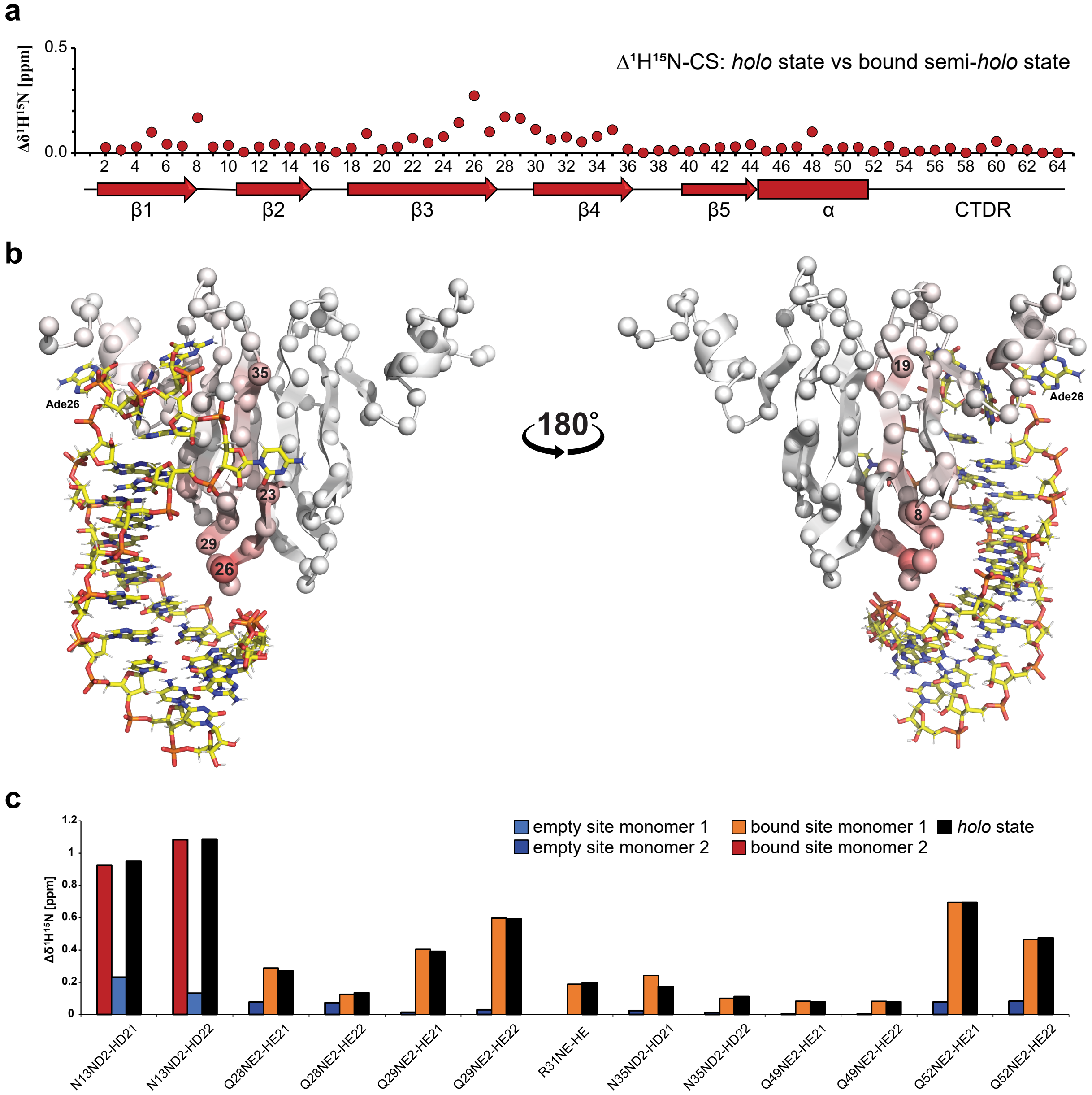

a, NH-CS differences between the bound site of the semi-*holo* SL2-RsmE dimer complex and the *holo* complex. The letter P indicates proline residues. **b**, NH-CSPs in panel *a* projected onto the semi-*holo* SL2-RsmE dimer complex shown for both the bound and empty sites. Intensity of red colour and diameters of spheres correlate with the magnitude of amide NH-CSP at every RsmE residue. **c**, Bar plot with the $^{1}H^{15}N$-CSP of side chain amides of the RsmE dimer upon binding to one (semi-*holo*) and two (*holo*) SL2 RNAs. Considering that the binding sites are composed of residues from both RsmE monomers, the bars from the semi-*holo* state are differently colour-coded for the two monomers (light and dark colours) and the two binding sites (blue/dark blue and red/orange colours).

Supplementary Figure 8: Increased ionic strength strongly reduces the binding affinity of first bound SL2 RNA for RsmE dimer.

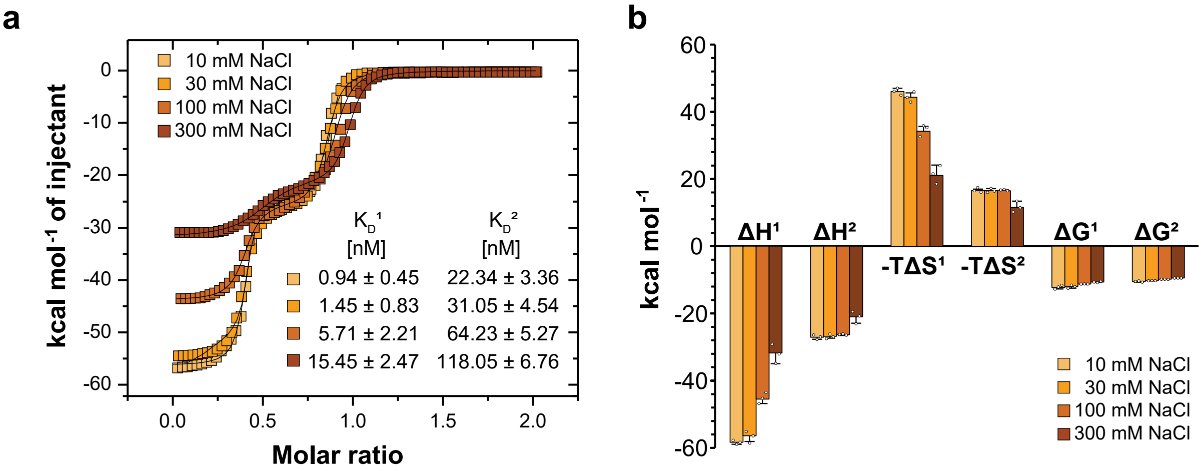

a, Overlaid ITC curves for the binding of SL2 RNA to the RsmE dimer with increasing concentration of NaCl in the buffer (50 mM potassium phosphate, pH 7.2). **b**, Bar plot with thermodynamic changes for the first and second binding events. Values are obtained from the average of three independent ITC titrations (shown data points) and errors are obtained from their standard deviation. Values obtained for individual titrations are shown in Supplementary Table 1.

Supplementary Figure 9: Comparison of chemical shift changes of backbone and side chain amides of RsmE dimer upon binding to SL2 and to 4bpSL2 RNA.

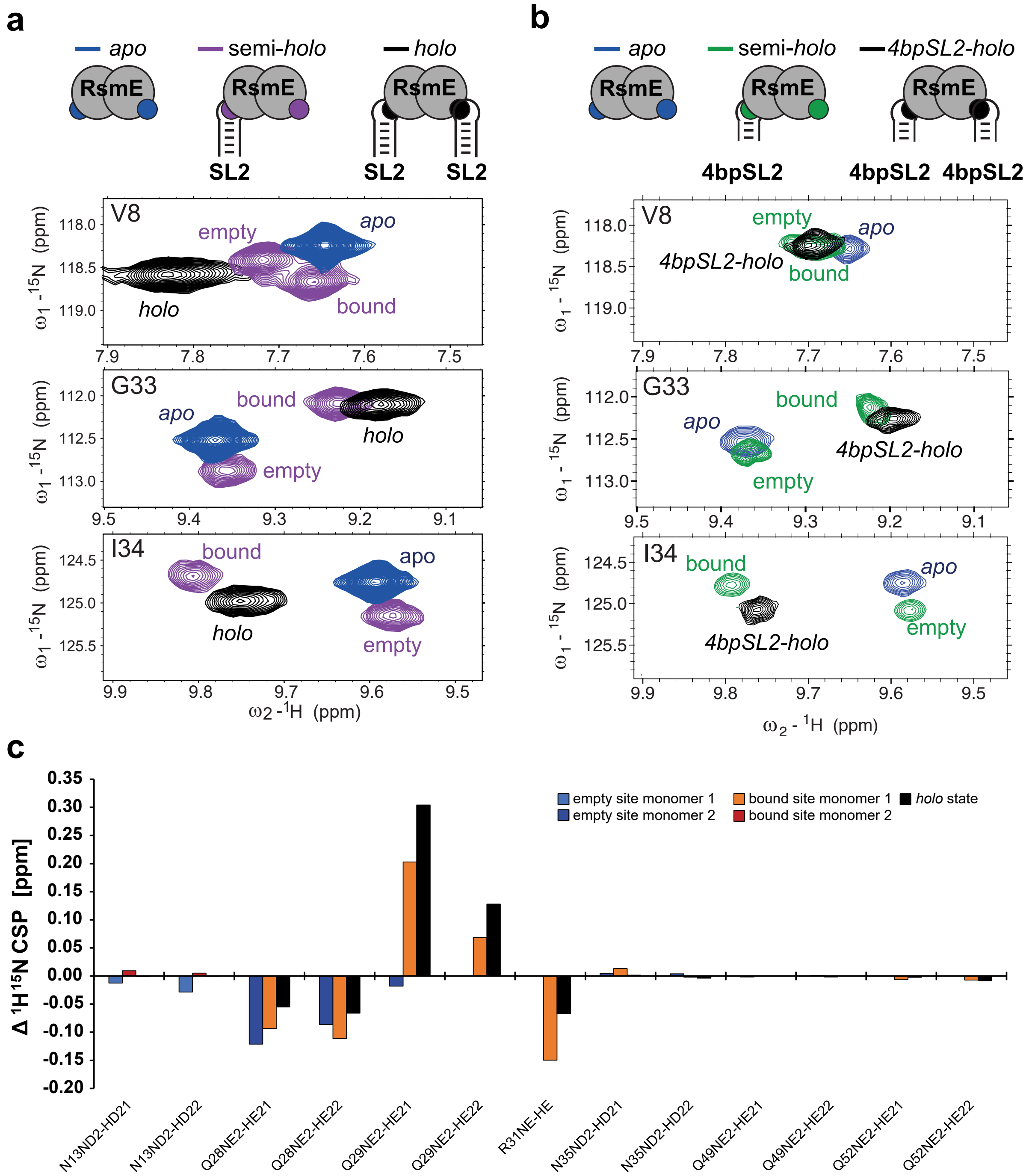

a, Three isolated backbone amide peaks in overlaid $^1H^{15}N$-HSQC spectra of the apo (blue), SL2 semi-*holo* (purple) and SL2 *holo* (black) ^{15}N-RsmE dimer states. **b,** Three isolated backbone amide peaks in overlaid $^1H^{15}N$-HSQC spectra of *apo* RsmE dimer (blue), 4bpSL2 semi-*holo* RsmE dimer in green and 4bpSL2 *holo* (black) RsmE dimer states. **c**, Bar plot showing the difference between the $^1H^{15}N$-CSP triggered by the binding of either 4bpSL2 RNA or SL2 RNA on the side chain amides of the RsmE dimer. Computed as follows: $\Delta\ ^1H^{15}N$ CSP = 4bpSL2-induced $^1H^{15}N$ CSP − SL2-induced $^1H^{15}N$ CSP. The same quantification was performed for the two *holo* states (Black bars).

Supplementary Figure 10: Line-broadening of backbone amide peaks in residues from the empty binding site of the RsmE dimer.

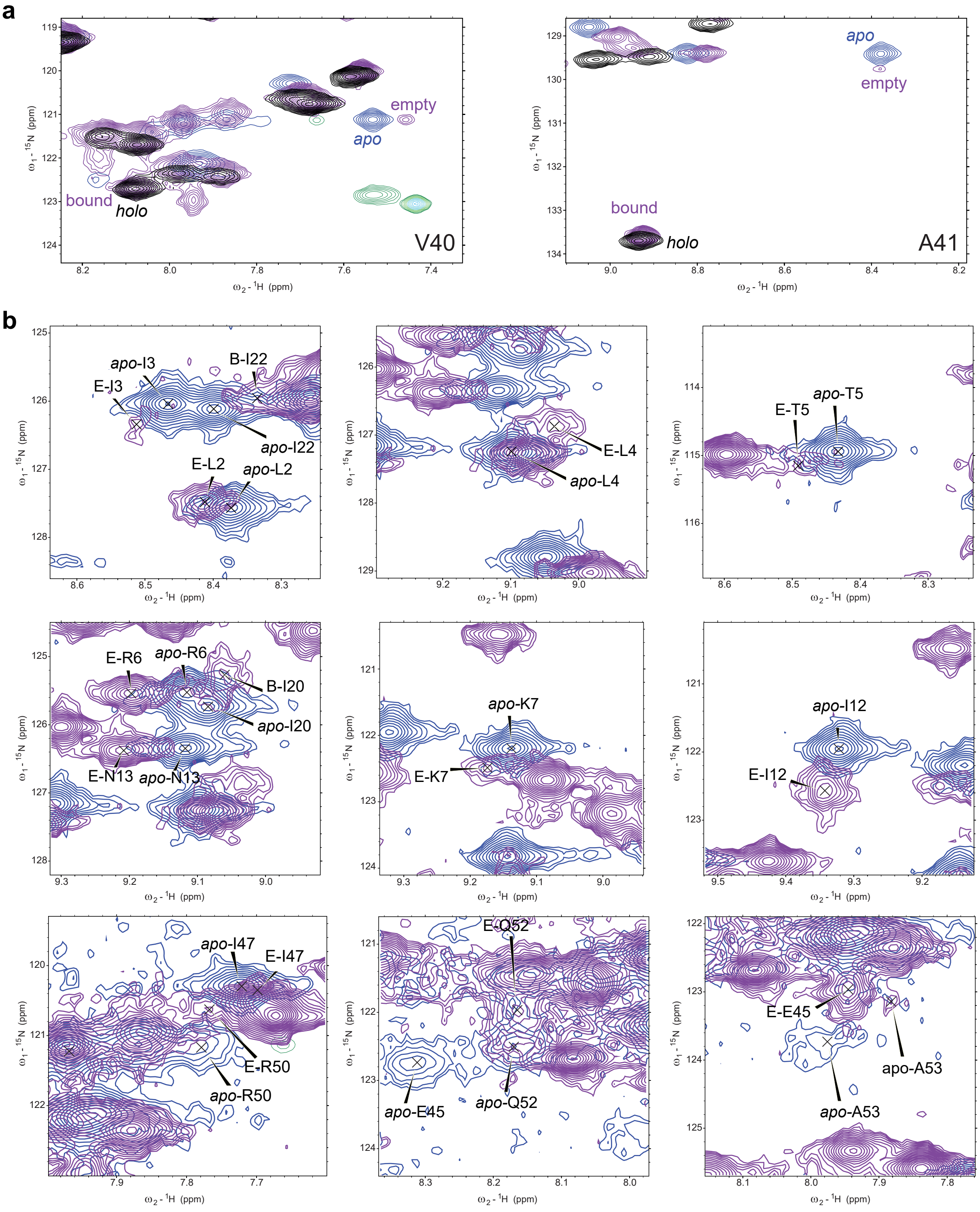

a, Overlay of V40 and A41 amide peaks in the $^{1}H^{15}N$-HSQC spectra of the RsmE dimer in its *apo* state (blue) and the semi-*holo* (purple) and *holo* (black) SL2-RsmE dimer. **b** Overlayed $^{1}H^{15}N$-HSQC spectral regions of the *apo* RsmE dimer (blue) and the semi-*holo* SL2-RsmE dimer complex (purple) for selected amide signals. Backbone amide peaks that experienced line-broadening are labelled.

Supplementary Figure 11: Upon binding to one SL2 RNA, some CS in the α-helix of the empty binding site of RsmE dimer move towards the random coil chemical shift.

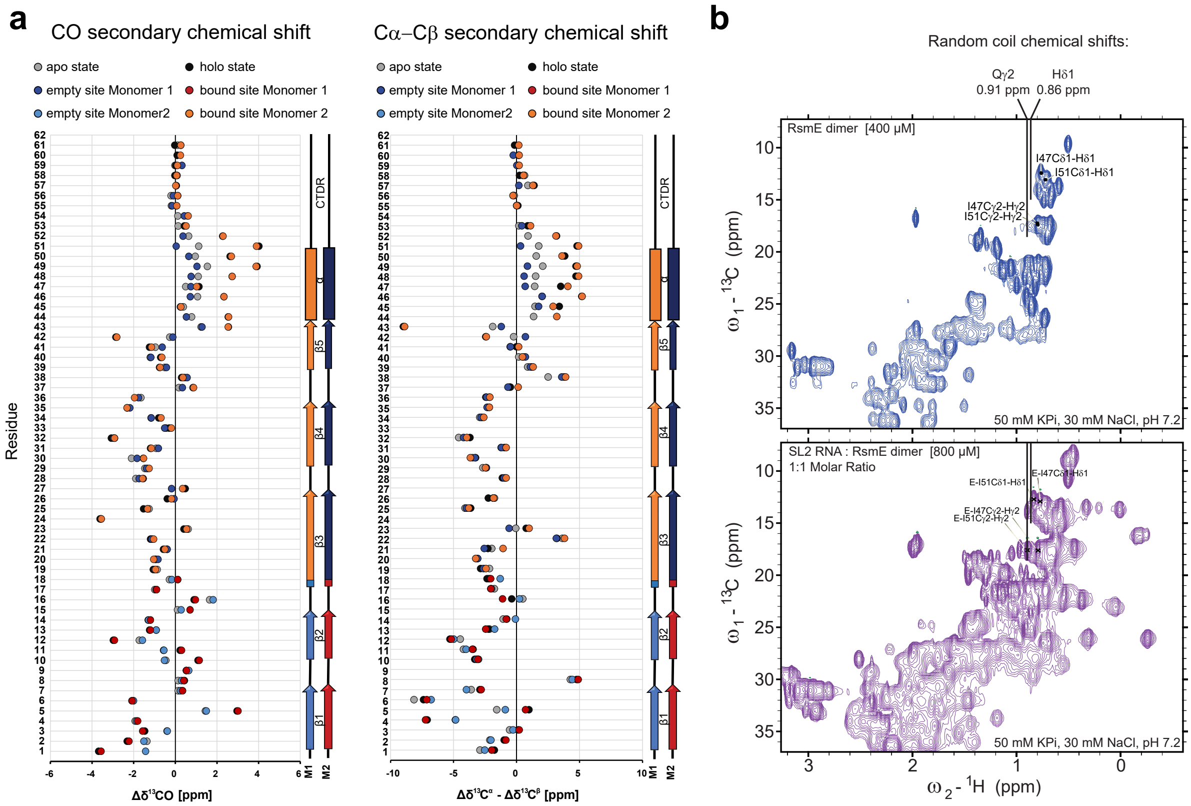

a, CO and Cα–Cβ chemical shift difference to the random coil CS in the *apo* RsmE dimer (grey), semi-*holo* SL2-RsmE complex (red, orange, light and dark blue) and *holo* complex (black). The differences from the semi-*holo* complex are colour-coded: warm colours (orange and red) correspond to residues on the bound site, cold colours (light blue and dark blue) refer to the empty site. The zero line (black) indicates the random coil chemical shifts. **b,** Methyl region of $^{1}H^{13}C$-HSQC spectra from the *apo* RsmE dimer (top, blue) and the semi-*holo* SL2-RsmE dimer (bottom, purple). Labels indicate I47 and I51 methyl (Hγ2 and Hδ1) peaks from the α-helix in the empty binding site of RsmE dimer. Random coil chemical shifts for Isoleucine methyl ^{1}H are indicated with black vertical lines.

Supplementary Figure 12: The α-helix of the empty binding site loses its contact with the β-sheets in the semi-*holo* SL2-RsmE dimer complex.

a

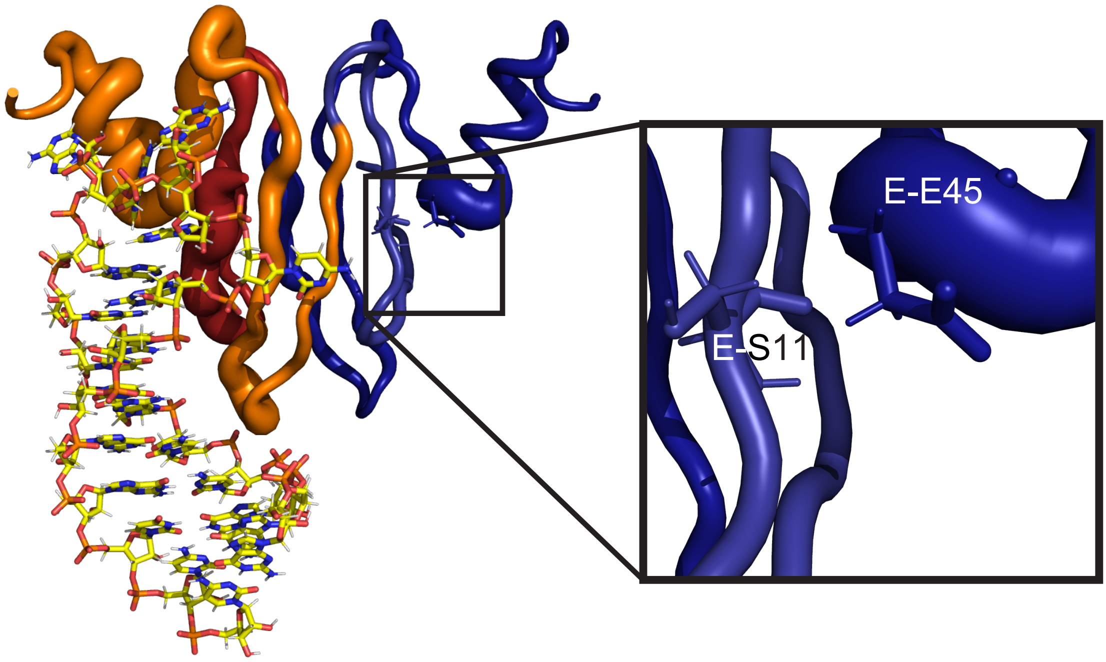

b

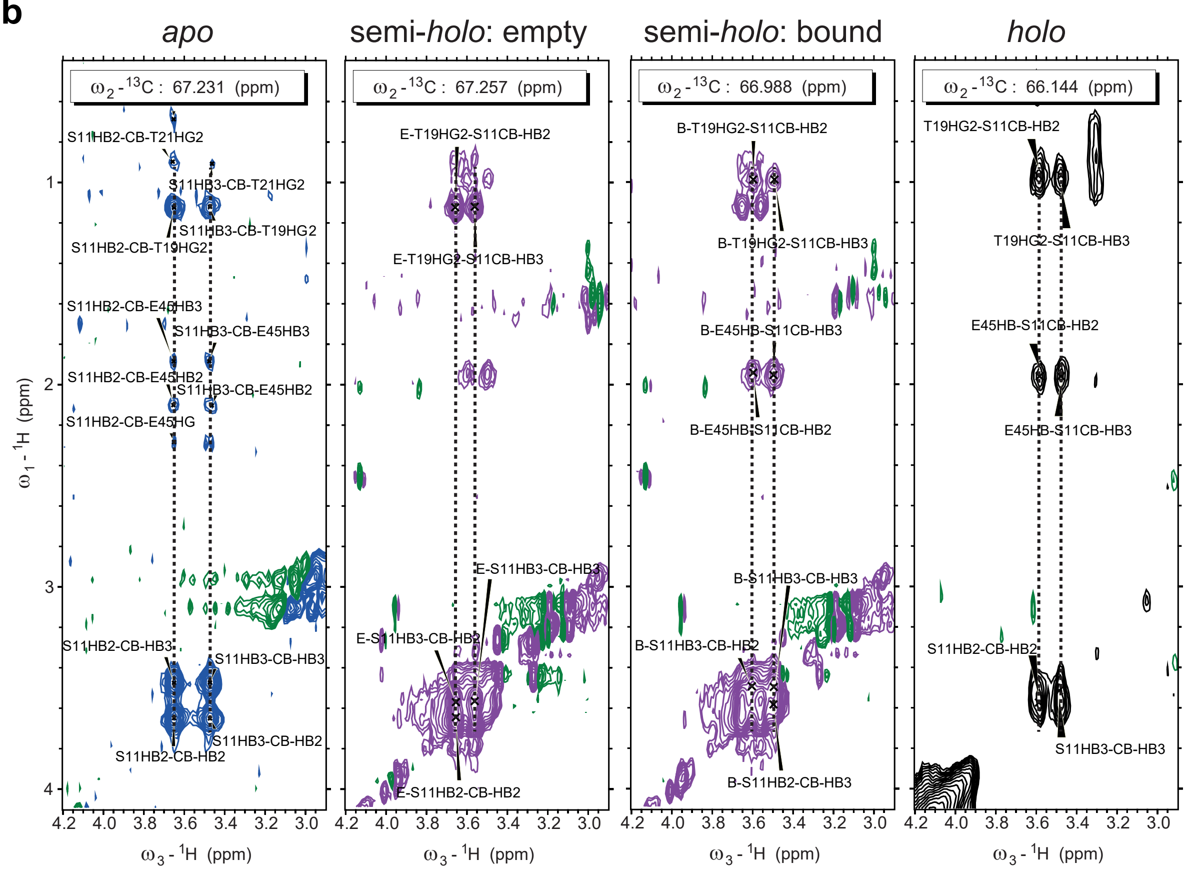

a, RsmE dimer depicted as a tube of variable width where tube thickness is proportional to NH CSPs. Contact between β-sheet and α-helix is mediated by residues S11 and E45. Coordinates for structure are derived from the SL2-RsmE dimer complex (PDB:2mfe). The sidechains of residues S11 and E45 in the empty site are shown. Insert shows an expanded view of these residues. **b**, Regions of the ^{13}C-resolved NOESY for the *apo*, semi-*holo*, and *holo* SL2-RsmE dimer complexes showing NOEs between S11 Cβ-Hβ2 and Hβ3 and E45Qβ protons.

Supplementary Figure 13: The MD simulations show increased dynamics in the empty site of semi-holo SL2 RNA-RsmE dimer complex.

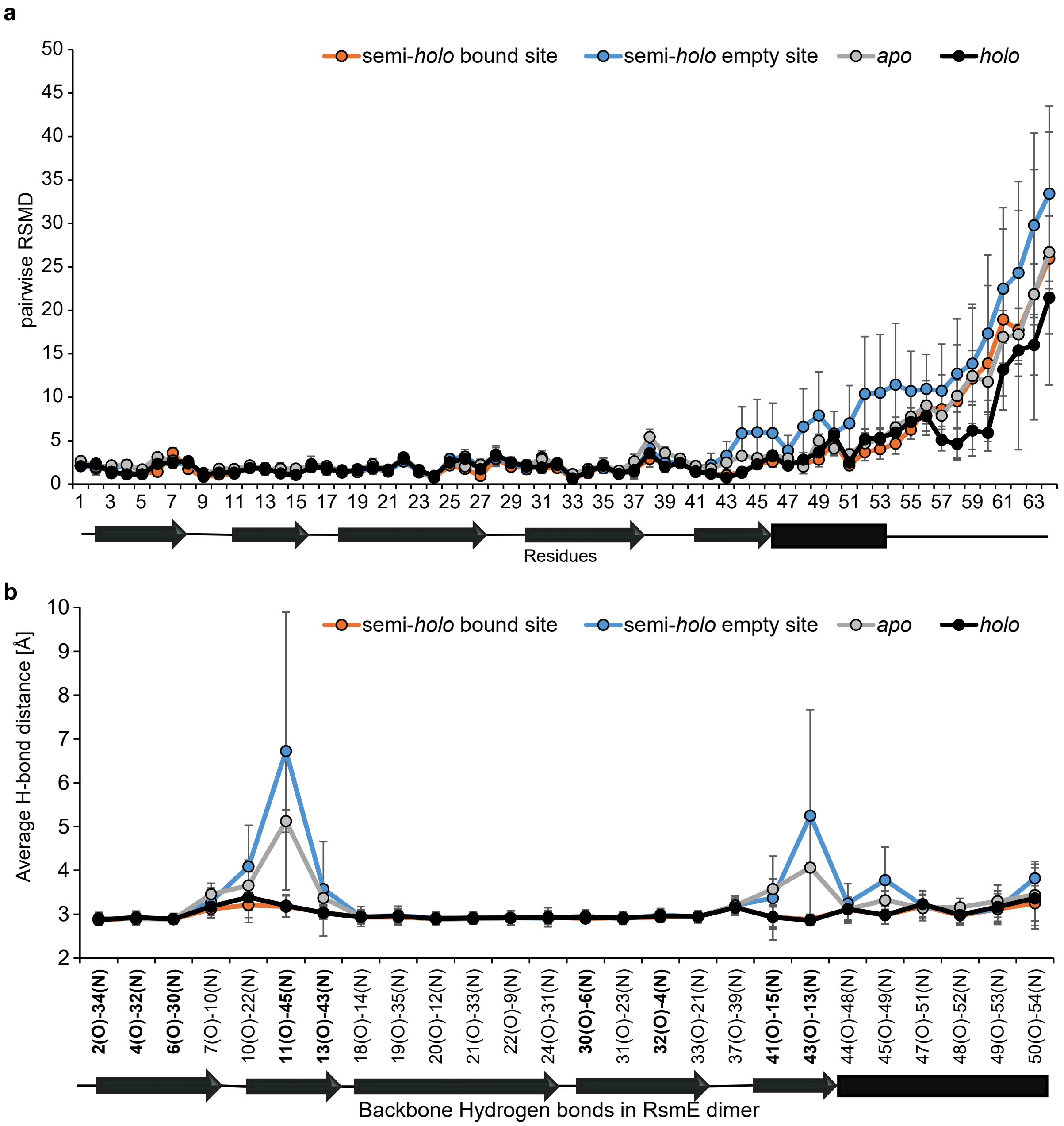

a, Pairwise Root Square Mean Deviation (pairwise RSMD) for backbone N atom of every residue in the RsmE dimer in the *apo*, semi-*holo* and *holo* states. Error bars correspond to the standard deviation of the pairwise RSMD throughout each set of MD simulations (40 μs total for each state). **b**, Average hydrogen bond partner distance in the backbone of RsmE dimer in the *apo*, semi-*holo* and *holo* states. Error bars indicate the standard deviation of the H-bond partner distance throughout each set of MD simulations. Intermonomeric H-bond pairs are highlighted in bold.

Supplementary Figure 14: The *apo* RsmE dimer undergoes a temperature-dependent structural transition.

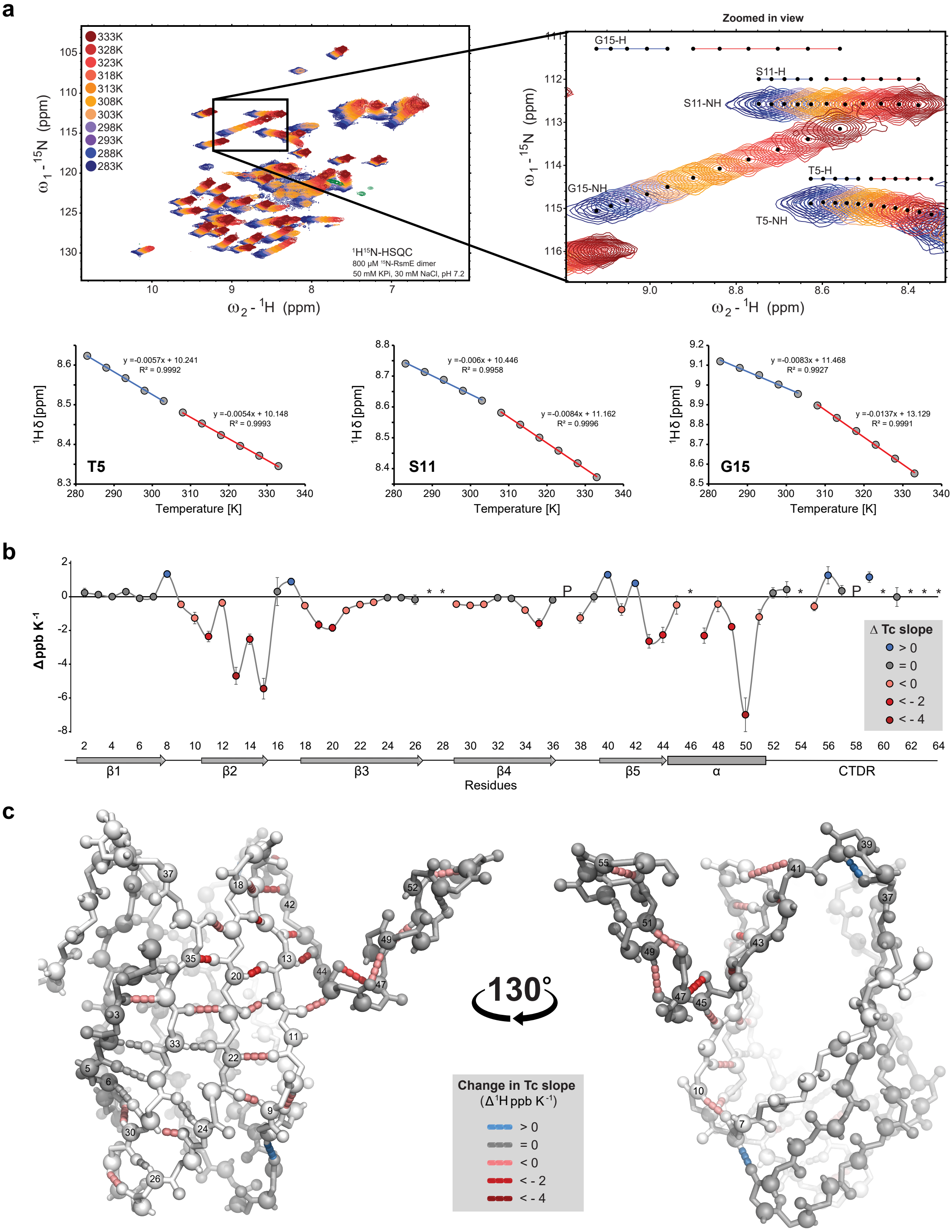

a, Overlaid $^1H^{15}N$-HSQC spectra of the RsmE dimer at different temperatures (283K to 333K). Three dot plots show amide proton CS temperature dependencies from three NH peaks. S11 and G15 showed non-linear temperature dependence with two linear regions at low and high temperature (temperature coefficients are given as Tc @<303K and Tc @ >308K). **b**, Dot and line plot indicating the changes in Tc (ΔTc = Tc @<303K – Tc @>308K in Δppb K^{-1}) plotted versus residue position in the *apo* RsmE dimer. Errors estimated from the linear fit of temperature dependence. A negative value corresponds to a more negative temperature coefficient for the conformation after the transition (> 303K). Stars (*) indicate backbone amides that are not observed at temperatures higher than 313K and therefore no change in slope could be calculated. The letter P marks proline residues. **c**, Cartoons of the backbone of the *apo* RsmE dimer. Monomers are differently coloured in light and dark grey. Segmented coloured lines correspond to hydrogen bonds that experienced non-linear temperature coefficients. The magnitude of the change in Tc is indicated by the colour scale.

Supplementary Figure 15: The binding of one SL2 RNA to RsmE dimer alters the hydrogen bonds in the β-sheets of RsmE dimer.

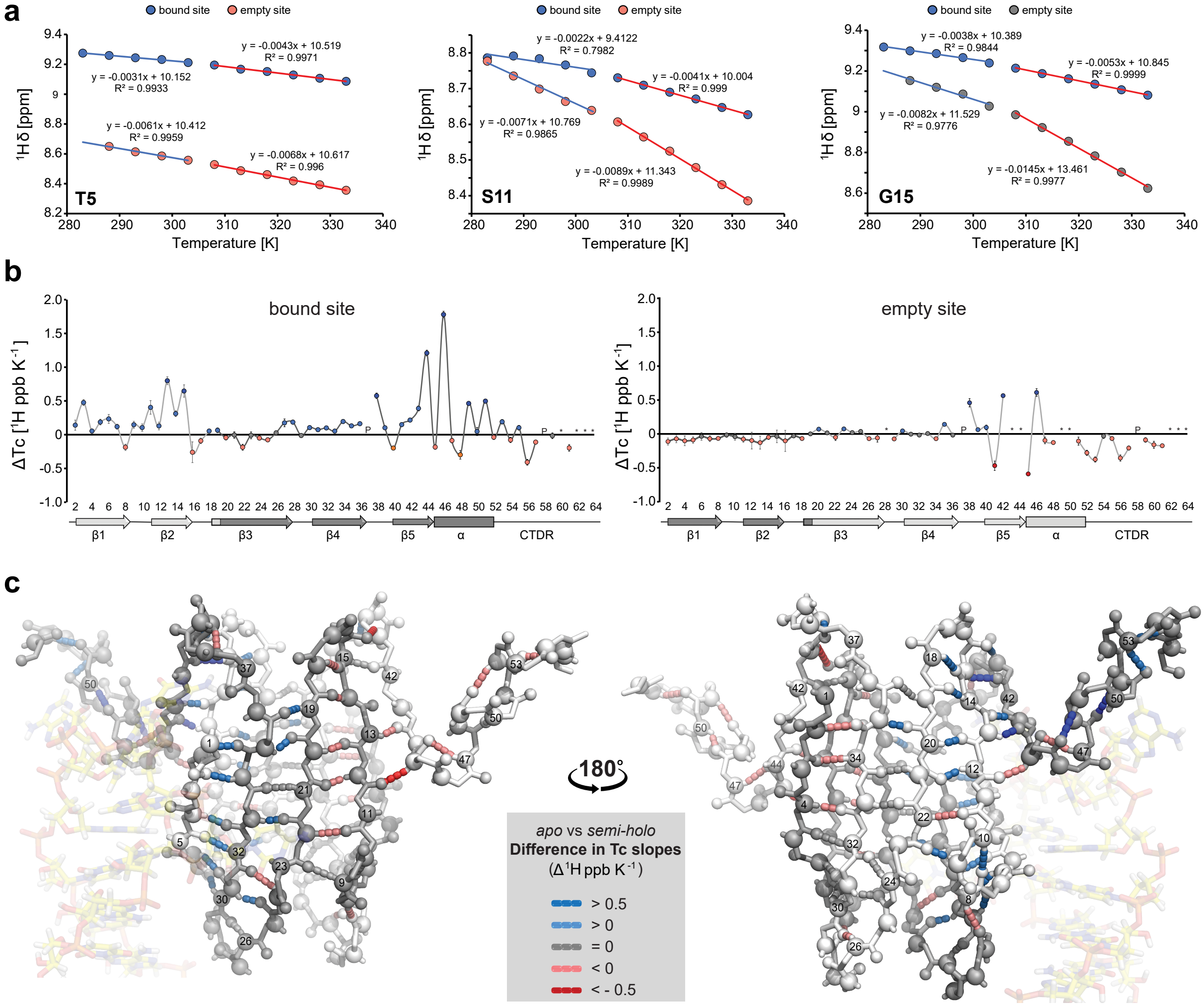

a, Three dot plots show amide proton CS temperature dependence of three H^N signals. They showed non-linear temperature dependence with two linear regions below 303K and above 308K. **b**, Two dot and line plots with the SL2 RNA-binding induced changes in temperature coefficient (ΔTc in ppb K^{-1}) plotted versus residue position for bound and empty sites of the RsmE dimer (semi-*holo* state). $\Delta Tc = [Tc@<303K_{semi\text{-}holo} + Tc@>308K_{semi\text{-}holo}] - [Tc@<303K_{apo} + Tc@>308K_{apo}]$. Errors estimated from linear fit of temperature dependence. A negative value corresponds to a more negative Tc for the semi-*holo* state. Stars (*) indicate backbone amides that are not observed at temperatures higher than 313K for which ΔTc could not be calculated. The letter P marks proline residues. **c**, Backbone atoms of the semi-*holo* SL2 RNA-RsmE dimer state. RNA is rendered transparent to ease RsmE dimer visualization. Monomers are differently coloured in light and dark grey. Interrupted coloured lines correspond to hydrogen bonds that experienced significant changes ($p<0.05$) in the Tc after binding of the first SL2 RNA. The magnitude of the change in Tc is indicated by the colour scale. Blue (larger ΔTc) correlates with a shortened H-bond and red (smaller or negative ΔTc) with a lengthening.

Supplementary Figure 16: The hydrogen-deuterium exchange curves of the backbone amides in the *apo* RsmE dimer and in the semi-*holo* SL2-RsmE dimer complex.

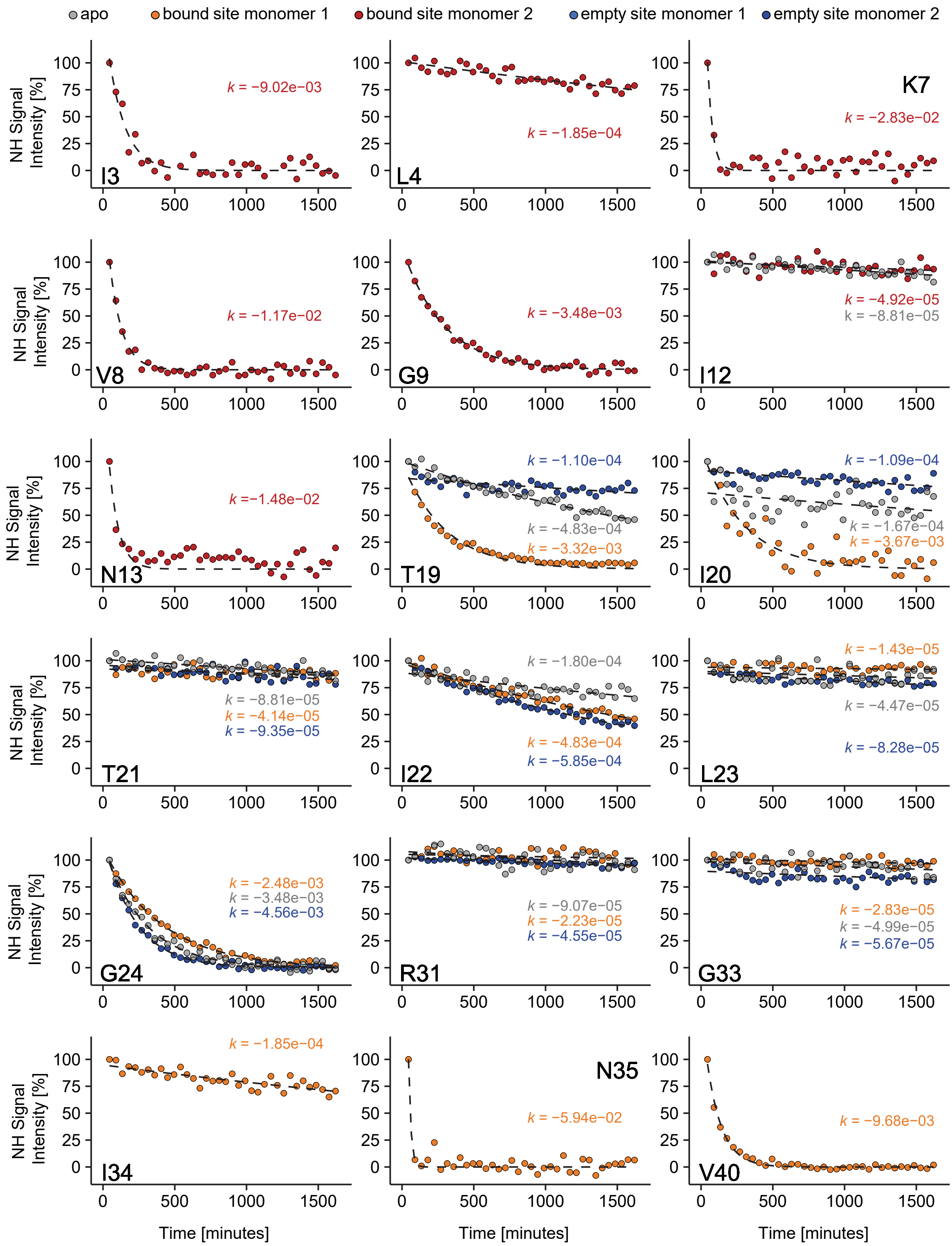

Observed HDX curves for well-resolved peaks of the backbone amides of the *apo* and semi-*holo* RsmE dimer states. Data are colour-coded following the colours in the RsmE monomer schemes in Figure 1,3 and 4. HDX curves of R6, E10, I14 and I32 are shown in Figure 5c. Fitted rate constants k are given in units of min^{-1}.

Supplementary Tables:

Supplementary Table 1: ITC parameters for binding of RsmE dimer to various RNAs.

Conditions		Experimentally determined values				Calculated values+			
RNA binder	NaCl in buffer*	K_D[1] [mean±SD, nM]	K_D[2] [mean±SD, nM]	ΔH[1] [mean, kcal/mol]	ΔH[2] [mean, kcal/mol]	−TΔS[1] [kcal/mol]	−TΔS[2] [kcal/mol]	ΔG[1] [kcal/mol]	ΔG[2] [kcal/mol]
SL2	10 mM	0.94±0.45	22.34±3.36	-58.33	-27.08	46.02	16.65	-12.31	-10.44
Repeat 1		1.25	20.14	-58.61	-27.41	46.49	16.82	-12.116	-10.587
Repeat 2		0.87	24.21	-58.36	-27.02	46.03	16.62	-12.333	-10.397
Repeat 3		0.71	22.68	-58.02	-26.82	45.52	16.49	-12.502	-10.331
SL2	30 mM	1.45±0.83	31.05±4.54	-56.38	-26.80	44.32	16.56	-12.06	-10.24
Repeat 1		2.02	33.91	-55.54	-27.06	43.68	16.87	-11.861	-10.189
Repeat 2		1.04	28.36	-57.24	-26.84	44.99	16.54	-12.254	-10.296
Repeat 3		1.29	30.88	-56.36	-26.51	44.23	16.27	-12.127	-10.245
SL2	100 mM	5.71±2.21	64.23±5.27	-45.47	-26.34	34.23	16.53	-11.25	-9.81
Repeat 1		5.45	63.75	-45.41	-26.33	35.58	16.51	-11.273	-9.816
Repeat 2		7.18	67.67	-44.16	-26.56	33.05	16.78	-11.110	-9.781
Repeat 3		4.51	61.27	-46.85	-26.12	35.47	16.28	-11.385	-9.839
SL2	300 mM	15.45±2.47	118.05±6.76	-31.75	-21.04	21.10	11.59	-10.66	-9.45
Repeat 1		15.29	117.69	-31.96	-20.91	21.30	11.46	-10.662	-9.453
Repeat 2		17.03	122.36	-30.06	-20.19	19.46	10.76	-10.598	-9.430
Repeat 3		14.02	114.11	-33.23	-22.03	22.52	12.56	-10.713	-9.471
4bpSL2	30 mM	21.56±4.18	97.23±5.84	-39.38	-23.34	28.93	13.77	-10.46	-9.57
Repeat 1		19.02	93.12	-40.23	-23.56	29.70	13.94	-10.532	-9.621
Repeat 2		24.14	99.62	-38.96	-23.12	28.57	13.60	-10.391	-9.521
Repeat 3		21.51	98.95	-38.96	-23.33	28.50	13.78	-10.460	-9.555
hcnA SD	30 mM	8.67±2.02	217.86±12.79	-38.92	-12.63	27.92	3.54	-10.998	-9.088
Repeat 1		6.37	203.15	-40.15	-13.08	28.97	3.95	-11.181	-9.129
Repeat 2		10.18	224.08	-38.54	-12.04	27.64	2.97	-10.903	-9.072
Repeat 3		9.45	226.36	-38.08	-12.76	27.13	3.69	-10.947	-9.066
hcnA SD▪	30 mM		297.09±11.43		-8.94		0.04		-8.91
Repeat 1			309.71		-9.13		0.25		-8.879
Repeat 2			287.43		-8.79		-0.13		-8.924
Repeat 3			294.14		-8.91		0.00		-8.911

+ ΔG values were calculated from the experimentally determined values by relating the association constant ($K_A = K_D^{-1}$), the experimental temperature (298.15 K) and the equilibrium constant (R) with the changes in the Gibbs free energy ($\Delta G=-RT\ ln\ K_A$). $T\Delta S$ values were calculated by subtracting the ΔG values from the ΔH values ($\Delta G= \Delta H- T\Delta S$). TΔS values are shown as $-T\Delta S$.

* The ITC buffer comprised 50 mM K_2HPO_4 at pH 7.2 and variable NaCl concentrations. Further details can be found in the Methods section.

▪ Titration of hcnA SD RNA into semi-*holo* SL2-RsmE dimer. The n-value for this set of single site titrations is shown in Supplementary Fig.1b.

Supplementary Table 2: MD simulations system setup.

System	Atoms	Box	Solvent molecules	Salt concentration	Lipids
Semiholo state	61729	Truncated Octahedron	19652	100 mM	None
Semiholo 2nd state*	49115	Truncated Octahedron	15442	100 mM	None
Apo state	69168	Truncated Octahedron	22368	100 mM	None
Holo state	71072	Truncated Octahedron	22514	100 mM	None

* Reported in Supplementary Figure 4.

Supplementary Notes:

Supplementary Notes 1: The binding of SL2 RNA to the SD-bound RsmE dimer causes chemical shift changes in both the RsmE-bound SD RNA and the SD-bound site of RsmE dimer.

The hcnA SD RNA is a stem-loop RNA that contains a SD sequence in its C**GGA** tetra-loop and is located 12 nucleotides upstream of the start codon of the hcnA mRNA. It binds the RsmE dimer with high affinity, and RsmE, therefore, is a strong repressor of hcnA mRNA expression[1]. We therefore explored whether the binding of SL2 RNA alters the high affinity binding of RsmE dimer to the hcnA SD RNA. As illustrated in the **Supplementary Figure 1a**, we first measured the binding curves for the hcnA SD RNA and RsmE dimer to determine their binding affinity, enthalpic and entropic terms using ITC. We also prepared a semi-*holo* complex using SL2 RNA and ^{15}N-labeled RsmE dimer, verified the semi-holo stoichiometry using NMR spectroscopy and determined the binding curve of hcnA SD RNA to the SL2 RNA-bound RsmE dimer. In the first ITC experiment (**Supplementary Figure 1b**), we could observe two distinct binding events in the binding curve with a much higher affinity in the first binding event than in the second, confirming that the high affinity binding of the hcnA SD RNA also triggered a negative allostery in the RsmE dimer. Importantly, we also observed that the binding affinity of hcnA SD RNA for RsmE dimer is further reduced when RsmE dimer was previously bound to a SL2 RNA from RsmZ ncRNA.

We next explored the effect of SL2 RNA binding to a hcnA SD-bound RsmE dimer using NMR spectroscopy. We first prepared a semi-holo hcnA SD-bound ^{15}N-labeled RsmE dimer sample and verified its semi-holo stoichiometry using NMR spectroscopy (**Supplementary Figure 1c**). The sample was then split into two fractions, and each fraction was saturated with either hcnA SD RNA or SL2 RNA to reach SD-holo and hetero-holo states, respectively. A third sample was also prepared with SL2 RNA and ^{15}N-labeled RsmE dimer to reach a SL2-holo state. The analysis of the ^{1}H,^{15}N-HSQC spectra from these three samples showed that high affinity second binder RNAs induced NH-CSPs in the previously SD-bound site of RsmE dimer (**Figure 1d and Supplementary Figure 1c**). Additionally, we recorded ^{1}H,^{1}H-TOCSY experiments of these NMR samples, which allowed us to assess whether the chemical shifts in the nine pyrimidines (H5-H6) in the RsmE-bound hcnA SD RNA exhibited CSPs upon binding of SL2 RNA to the other binding site of RsmE dimer. We could observe that most of the H5-H6 signals in the RsmE-bound hcnA SD RNA experienced CSPs upon SL2 RNA binding to the empty site of RsmE dimer (**Figure 1e**). The complete assignments (**Supplementary Figure 1d**) of these chemical shifts were previously reported by Schubert et al and Duss et al[1,7].

Supplementary Note 2: On the assignment and quantification of the semi-*holo* RsmE states.

To assign the chemical shifts (CS) in the *apo* and semi-*holo* ^{15}N-RsmE states, we first recorded and analysed backbone-assignment (HNCACB, HNCA, HNCO, HN(CO)CA and HN(CA)CO) and side-chain assignment (3D HcCH-TOCSY) experiments at 313K. To assign some NH-CS that showed broad linewidth, backbone assignment experiments were also recorded at 305K and 308K. This approach allowed us to completely assign the *apo* RsmE state and partially assign the main semi-*holo* states (states I and II). Considering that we have previously assigned the *holo* RsmE state[1], we assigned the remaining CS by comparing ^{1}H^{15}N-HSQC and backbone assignment experiments to the corresponding spectra of the *apo* and *holo* RsmE dimers. Moreover, we recorded 3-dimensional ^{15}N- and ^{13}C-resolved NOESY experiments using long mixing times (150 ms) to confirm, using through space correlations, the independent assignment of both monomers in the semi-*holo* states.

To assign the additional states in the bound site of the semi-*holo* SL2 RNA-RsmE dimer complex, we first observed that one of the additional peaks in the ^{1}H^{15}N-HSQC spectrum has a similar CS to the B-E46-NH (**Supplementary Figure 3a**), which underwent a large CS perturbation upon RNA binding. We also observed that this additional NH peak was associated with Cα (**Supplementary Figure 3b**) chemical shifts that are similar to those in B-E46-NH. We then followed a sequential walk in the ^{15}N-resolved NOESY (**Supplementary Figure 3c**) supplemented by backbone assignment experiments (HNCA and HNCO) to assign the backbone of the additional state II in the bound CTDR and α-helix. We then used the assigned HN, Cα, and Hα CS as a starting point in the ^{15}N- and ^{13}C-resolved NOESY and the 3D HcCH-TOCSY spectra to assign the sidechain signals of the additional state II. Despite the low abundance of the additional state III, resonances were identified from their similarity with state II correlations for sidechains in the 3D HcCH-TOCSY and ^{13}C-resolved NOESY spectra. We therefore refer to these state III assignments as tentative.

To quantify the abundance of the three states observed for the CTDR and α-helix of the semi *holo* state of RsmE dimer, we integrated the ^{1}H^{13}C-HSQC peak volumes for isolated methyl groups from these structural elements of the RsmE dimer and we also measured the peak intensity of non-isolated ^{1}H^{13}C-HSQC peaks. These two approaches yielded similar results. In the main text, the results of the first approach are provided.

Supplementary Note 3: Additional MD simulations with truncated RsmE C-terminus. (Related to Figure 2 and Supplementary Figure 4)

To sample more structural exchange events between the two CTDR states during the same μs MD timescale, we performed additional simulations of the semi-holo state using a truncated RsmE C-terminus. The CTDR in these simulations was truncated to four residues

(RsmE residues 56 to 59), instead of 14 residues. Residues 56 to 59 were kept because residues 57 and 58 were experimentally shown to contact Ade26[1]. We hypothesized that, by reducing the length of the CTDR, this disordered region would be able to navigate faster through the solvent. We performed two additional sets of three MD simulations in explicit solvent. They differed from each other only in the starting conformation (**Supplementary Figure 4b**); one set started from the same starting conformation as in the initial four simulations in Supplementary Fig.3a (the Ade26-bound CTDR conformation), and the other set started from the additional conformation that was observed in the fourth of the initial MD simulations (the Ade26-detached CTDR conformation. In these simulations, we indeed observed an increase in CTDR-detachment events as well as emergence of the CTDR conformation that was used for the second set of CTDR-truncated MD simulations. On the other hand, in the Ade26-detached CTDR simulations, we observed that MD state II was quite stable on the same μs MD timescale.

Supplementary Note 4: On the fractional sodium occupancy in MD simulations of SL2 RNA-RsmE dimer. (Related to Figure 3 and Supplementary Figure 6a)

To investigate whether sodium ion bridges could mediate the interaction between the SL2 RNA stem and the β3-β4 loops of RsmE dimer, a volumetric map at 1 Å resolution was built for the fractional occupancy of sodium ions throughout the MD simulations, using the VolMap tool in the VMD software. A volumetric map is a 3D grid that contains a value at each grid point. After aligning the backbone of the RsmE dimer in the centre of the 3D grid throughout the MD simulation, each grid point (1 $Å^3$ volume cube) in each MD frame was set to either 0 or 1, depending on whether the grid point was "occupied" by a sodium ion or not. By averaging over all frames, the fractional sodium occupancy was calculated for each grid point. Thus, in the SL2 RNA-RsmE dimer MD simulation, the averaged fractional sodium occupancy in the explicit solvent was < 0.0005. The averaged fractional sodium occupancy shielding the RsmE-SL2 complex was < 0.005. The volumetric map in the left panel of **Supplementary Figure 6a** is shown at a cutoff of 0.05, i.e., each green volume corresponds to grid points with a fractional sodium occupancy larger than 0.05. This approach allowed us to identify clusters of high fractional sodium occupancy. Importantly, the grid points in the sodium cluster in proximity to β3-β4 loops (Shown in Figure 3a in the main text) have fractional sodium occupancies ranging from 0.08 to 0.10, corresponding to a ~200× locally elevated sodium concentration compared to the bulk.

Supplementary Note 5: Evidence for partial unfolding of the empty α-helix in the semi-*holo* SL2 RNA-RsmE dimer complex. (Related to Figure 4 and Supplementary Figure 11)

Upon binding the first SL2, three amide peaks of the RsmE dimer in the first helical turn of the empty site α-helix (E-R44, E-E45 and E-E46) moved toward the centre of the $^{1}H^{15}N$-HSQC

spectrum. Considering that the α-helix is extensively involved in RNA recognition, we speculated that these changes might indicate a partial unfolding of the α-helix, which can lead to an impaired RNA recognition and reduced affinity of the second binding event. Secondary Cα, Cβ and CO CS report on changes in average secondary structure[2,3]. Upon binding of one SL2 RNA, several CO shifts in the empty α-helix of RsmE dimer showed smaller secondary shifts than in the *apo* RsmE dimer, suggesting a shift toward a less folded form in the folded-unfolded equilibrium (Left-side panel in **Supplementary Figure 11a**). Secondary Cα-Cβ shifts supported this interpretation (Right-side panel in **Supplementary Figure 11a**).

Another NMR feature of a random coil conformation is the sharpening of $^{1}H^{13}C$ peaks in the $^{1}H,^{13}C$-HSQC. As opposed to amide peaks, carbon-attached hydrogen protons do not chemically exchange with the solvent, and their chemical shift and peak line-shape are mainly determined by their chemical environment and their correlation time (τ_c). In a random coil conformation, amino acids show a shorter τ_c, with slower T2 relaxation and narrower line shape. The inspection of line-shape in peaks from methyl groups (I47H 2, I47H 1, I51H 2 and I47H 1) showed sharp peaks in the empty α-helix of the RsmE dimer when it is bound to one SL2 RNA. These methyl groups also changed their chemical shifts, as compared to the *apo* RsmE dimer. Their ^{1}H chemical shifts approached their corresponding RC chemical shift values (**Supplementary Figure 11b**). Taken together, the backbone carbon secondary shift quantification and the analysis of methyl group chemical shifts confirmed that, in the semi-*holo* state, the empty α-helix undergoes a partial unfolding.

Importantly, some residues in the *apo* state, e.g. E45, E46, I47 and R50, also showed broad linewidths in the $^{1}H^{15}N$-HSQC spectrum (**Supplementary Figure 10b**). These residues map to the hinge region between the β5 strand and the α-helix, and to the first helical turn of the α-helix, suggesting that the α-helix undergoes conformational exchange, with an intermediate exchange regime, between a low and a high energy state in the μs-ms timescale. We can therefore infer that the observed changes in the empty α-helix of the semi-*holo* complex represents a shift of the folded-unfolded equilibrium towards a partially unfolded state.

To assess how the helical fold can be perturbed by binding of one SL2 RNA, we recorded ^{13}C-resolved NOESY experiments on the *apo* ^{13}C-RsmE dimer and on the ^{13}C-RsmE dimer bound to one or two SL2 RNA and searched for contacts between the α-helix and the β-sheets of the RsmE dimer. Clear NOEs between E45 in the first helical turn and S11 in the β2-strand were observed in the ^{13}C-resolved NOESY spectra of the *apo* RsmE dimer and SL2-saturated *holo*

RsmE dimer (**Supplementary Figure 12**). In the NOESY spectrum of the semi-*holo* RsmE dimer, the same NOEs were observed on the bound α-helix. However, these NOEs were absent for the empty site, suggesting that the partial unfolding of the empty α-helix, is also associated with a loss of its contact with the β-sheet of the RsmE dimer.

Supplementary Note 6: An *in-silico* investigation into the dynamic changes in the empty binding site of the semi-*holo* SL2 RNA-RsmE dimer complex.

To assess whether the experimentally observed dynamic changes in the empty binding site of the semi-*holo* complex were also observable in the MD simulations, we compared the pairwise Root Mean Squared Deviation (pRMSD) for the backbone N atoms and the backbone NH-CO Hydrogen bond partner distances throughout the 400,000 frames in each set of MD simulations (*apo*, semi-*holo* and *holo* states). Larger pRSMD values (**Supplementary Figure 13a**), an indicator of enhanced dynamics, were detected in the β5-strand and the α-helix of the empty binding site of the semi-*holo* complex, when compared to the pRMSD in the *apo* state. These enhanced dynamics in β5 and the α-helix were also associated with larger H-bond distances for the backbone H-bonding partners connecting the β2- and β5-strands in the empty side of the semi-*holo* SL2-RsmE dimer complex (**Supplementary Figure 13b**). These two *in-silico* observations are consistent with the experimental NMR data discussed in **Supplementary Note 5** and the lengthening of H-bonds reported by the amide temperature coefficients (**Figure 5**). Conversely, the MD simulations showed no increased dynamics in the β1-strand of the empty site of the semi-*holo* complex. This discrepancy may come about due to the inherent technical limitations of MD simulations, such as force field imperfections and the limited timescales probed. It can also result from the use of a SL2-bound RsmE state as the starting conformation for all MD simulations. This starting conformation may introduce some biases by locking in structural elements to a local minimum, therefore limiting the conformational space that the structure may explore during the MD simulations. Some of these biases are likely to persist during the production simulations and cannot be eliminated without an experimentally determined structure of the semi-holo SL2–RsmE complex.
