# Supplementary material for: Newton’s cradle-like allosteric mechanism explains regulatory RsmE RNA binding
Source: Nat Commun. 2026 Apr 22;17:5545. doi: 10.1038/s41467-026-72126-z (PMC13287468; doi:10.1038/s41467-026-72126-z)
Supplement: Supplementary file 2 — Reporting Summary [file 41467_2026_72126_MOESM2_ESM.pdf]

nature portfolio

Corresponding author(s): Frédéric H.-T. Allain

Last updated by author(s): Apr 2, 2026

Reporting Summary

Nature Portfolio wishes to improve the reproducibility of the work that we publish. This form provides structure for consistency and transparency in reporting. For further information on Nature Portfolio policies, see our Editorial Policies and the Editorial Policy Checklist.

Statistics

For all statistical analyses, confirm that the following items are present in the figure legend, table legend, main text, or Methods section.

n/a	Confirmed	
☐	☒	The exact sample size (*n*) for each experimental group/condition, given as a discrete number and unit of measurement
☐	☒	A statement on whether measurements were taken from distinct samples or whether the same sample was measured repeatedly
☒	☐	The statistical test(s) used AND whether they are one- or two-sided<br>*Only common tests should be described solely by name; describe more complex techniques in the Methods section.*
☒	☐	A description of all covariates tested
☒	☐	A description of any assumptions or corrections, such as tests of normality and adjustment for multiple comparisons
☐	☒	A full description of the statistical parameters including central tendency (e.g. means) or other basic estimates (e.g. regression coefficient) AND variation (e.g. standard deviation) or associated estimates of uncertainty (e.g. confidence intervals)
☒	☐	For null hypothesis testing, the test statistic (e.g. *F*, *t*, *r*) with confidence intervals, effect sizes, degrees of freedom and *P* value noted<br>*Give P values as exact values whenever suitable.*
☒	☐	For Bayesian analysis, information on the choice of priors and Markov chain Monte Carlo settings
☒	☐	For hierarchical and complex designs, identification of the appropriate level for tests and full reporting of outcomes
☒	☐	Estimates of effect sizes (e.g. Cohen's *d*, Pearson's *r*), indicating how they were calculated

Our web collection on statistics for biologists contains articles on many of the points above.

Software and code

Policy information about availability of computer code

Data collection	NMR data were measured and processed with Topspin3.6 or Topspin4.0. ITC data was collected and analysed with Microcal Origin 7.0 software.
Data analysis	Heteronuclear NOEs analysed with DynamicsCenter 2.5. NH temperature coefficients were fitted in Excel and HDX exchange fitted with R 5.4. Amide exchange plots were generated with R using R script and R data source files.<br>NMR spectra were analysed with NMRFAM-SPARKY 1.4. MD was performed with Amber18, and trajectories analysed with its cpptraj module.

For manuscripts utilizing custom algorithms or software that are central to the research but not yet described in published literature, software must be made available to editors and reviewers. We strongly encourage code deposition in a community repository (e.g. GitHub). See the Nature Portfolio guidelines for submitting code & software for further information.

Data

Policy information about availability of data

All manuscripts must include a data availability statement. This statement should provide the following information, where applicable:

- Accession codes, unique identifiers, or web links for publicly available datasets
- A description of any restrictions on data availability
- For clinical datasets or third party data, please ensure that the statement adheres to our policy

All data necessary to support the conclusions of this study are provided in the main text, main figures, Supplementary Information, and Source data files. NMR spectra, and ITC data files, used for quantification are available from the corresponding author upon request. MD trajectories used for quantification have been

deposited at MDrepo MDR00020893 [https://mdrepo.org/explore/20893], MDR00020894 [https://mdrepo.org/explore/20894], MDR00020895 [https://mdrepo.org/explore/20895], MDR00020896 [https://mdrepo.org/explore/20896], MDR00020897 [https://mdrepo.org/explore/20897], MDR00020898 [https://mdrepo.org/explore/20898], MDR00020899 [https://mdrepo.org/explore/20899], MDR00020900 [https://mdrepo.org/explore/20900]) and starting coordinates for MD runs at Zenodo with accession code 18334758 [https://doi.org/10.5281/zenodo.18334758]. A source data file for this paper is also available at Zenodo under accession code 19081039 [https://doi.org/10.5281/zenodo.19081039]. The coordinates for the holo dimer of RsmE used to model the semi-holo complex are available from the protein databank under accession code 2MFE [http://doi.org/10.2210/pdb2mfe/pdb]. The chemical shifts used as a starting point for assigning signals in the semi-holo state are available from the biological magnetic resonance databank under accession code 19546 [https://dx.doi.org/10.13018/BMR19546]. Chemical shifts obtained for the apo state at 303K and 313K, and for the semi-holo state at 313K have been deposited at the BMRB databank with accession codes BMRB 53701 [https://dx.doi.org/10.13018/BMR53701] (apo 303K), BMRB 53702 [https://dx.doi.org/10.13018/BMR53702] (apo 313K), and BMRB 53703 [https://dx.doi.org/10.13018/BMR53703] (semi-holo), respectively.

Research involving human participants, their data, or biological material

Policy information about studies with human participants or human data. See also policy information about sex, gender (identity/presentation), and sexual orientation and race, ethnicity and racism.

Reporting on sex and gender	N/A
Reporting on race, ethnicity, or other socially relevant groupings	N/A
Population characteristics	N/A
Recruitment	N/A
Ethics oversight	N/A

Note that full information on the approval of the study protocol must also be provided in the manuscript.

Field-specific reporting

Please select the one below that is the best fit for your research. If you are not sure, read the appropriate sections before making your selection.

[x] Life sciences [ ] Behavioural & social sciences [ ] Ecological, evolutionary & environmental sciences

For a reference copy of the document with all sections, see nature.com/documents/nr-reporting-summary-flat.pdf

Life sciences study design

All studies must disclose on these points even when the disclosure is negative.

Sample size	Sample sizes for ITC, NMR, and MD are necessarily small due to the large cost, and time required for repetitions. The number of repetitions are indicated where appropriate in methods, figure captions, and Source Data.
Data exclusions	No data were excluded.
Replication	ITC were repeated multiple times as indicated in supplementary Table 1 and were reproducible. NMR spectra were generally reproducible for different samples under the same conditions.
Randomization	Not applicable.
Blinding	Not applicable.

Reporting for specific materials, systems and methods

We require information from authors about some types of materials, experimental systems and methods used in many studies. Here, indicate whether each material, system or method listed is relevant to your study. If you are not sure if a list item applies to your research, read the appropriate section before selecting a response.

Materials & experimental systems

n/a	Involved in the study
☒	☐ Antibodies
☒	☐ Eukaryotic cell lines
☒	☐ Palaeontology and archaeology
☒	☐ Animals and other organisms
☒	☐ Clinical data
☒	☐ Dual use research of concern
☒	☐ Plants

Methods

n/a	Involved in the study
☒	☐ ChIP-seq
☒	☐ Flow cytometry
☒	☐ MRI-based neuroimaging

Plants

Seed stocks	N/A
Novel plant genotypes	N/A
Authentication	N/A
