# Supplementary material for: Newton’s cradle-like allosteric mechanism explains regulatory RsmE RNA binding
Source: Nat Commun. 2026 Apr 22;17:5545. doi: 10.1038/s41467-026-72126-z (PMC13287468; doi:10.1038/s41467-026-72126-z)
Supplement: Supplementary file 3 — Transparent Peer Review File [file 41467_2026_72126_MOESM3_ESM.pdf]

nature portfolio

Peer Review File

Newton’s cradle-like allosteric mechanism explains regulatory RsmE RNA binding

Corresponding Author: Professor Frédéric Allain

This file contains all reviewer reports in order by version, followed by all author rebuttals in order by version.

Version 0:

Reviewer comments:

Reviewer #1

(Remarks to the Author)
In the submitted manuscript, Finol and colleagues present a thorough NMR- and MD-based study targeting the molecular details of negative cooperativity in RsmZ RNA-binding by the dimeric RsmE protein. This process is utilized by bacteria, where the ncRNA RsmZ acts as a sponge to remove RsmE from mRNAs’ SD sequences and thereby enabling translation of the mRNA. The effective hand-over of RsmE from its two unequal binding sites around the SD sequence to neighbouring stem-loops in RsmZ has remained incompletely understood, also in light of a relatively braod range of possible affinities between RsmE monomers and the respective GGA motifs in the mRNA or RsmZ.
The authors here use ITC together with NMR CSP, NOESY and relaxation data and MD simulations to suggest a model (in analogy to Newton’s cradle), in which they suggest that changes in conformational entropy along the two binding sites in the RsmE dimer account for the modulation of affinity for RNA in the stepwise binding process. This is solidly corroborated by relaxation and H/D exchange data, guided by MD simulations suggesting multiple bound states.

The manuscript addresses a non-trivial question and uses very detailed insights into a protein-RNA system, which is quite well understood on the structural side, while mechanistic details have remained incompletely explored. The presented data are in principle trustworthy, well-presented and complementary, i.e. they yield a robust set of results to feed the mechanistic model. The text is also well written, and the manuscript overall concise and straightforward.
I feel the story does deserve to be published in this journal, as it manages to yield insight into fine details for how cooperativity works in this way, something that may also help to push similar systems by the choice of proper methods. There is little I have to criticize about the given data, but I would need a bit of add-on to support the model with suitable experimental data. I think this will strengthen the arguments and make the story be rounded up.

These are my concrete points:
- Regarding the model: In what is shown in Fig. 6 binding of RsmZ SL3 competes with binding of the RsmE subunit to the SD SL motif within the mRNA. From the previously published numbers this appears to be not so likely. I find the suggested model justified, but I wonder if the authors can provide experimental evidence supporting this mechanism, e.g. in designing a competing binding experiment with the relevant players? Can the effect of SL2-binding towards the affinity for the SD SL in the other monomer be shown or quantified? How much would this be decreased to allow unbinding?
- Also, the authors claim there is no cooperativity with only weak binders. Is this supported by experimental data, e.g. by H/D exchange or unfolding studies in response to RsmZ SL3?
- Adding to the above: The data on cooperativity have been obtained with SL2 as the readout, which makes sense. However, in the model the relevant relative affinities and thus effects apparently have to take into consideration initial one-site saturation with SL2, and then SL3! Are concentrations of RsmE and RsmZ in the cell known? Could an alternative model be, that two RsmZ RNAs remove RsmE as a dimer through their individual SL2s?
- All experiments assume that a SL2-RsmE dimer ratio of 1:1 leads to exclusive saturation of the high-affinity monomer. But seeing the numbers from ITC there should be a mentionable amount of two-site binding (statistically). I do not question the numbers obtained from experiments, but I suggest to mention this in the text, and ideally quantify (at least theoretically) all given states in the semi-holo complex.
- Just for a discussion: could there also be cooperative effects within the RNA SLs of RsmZ?

As minor issues:
- ITC data should be shown with all repetitions and error bars be added to the plots of thermodynamic parameters (e.g. Fig. 1a). Also, n-values should be given (molar ratios).
- Please provide the comparison of spectral overlays showing SL2 vs. 4bpSL2 titrations!
- Page 5, Fig. 2e: how exactly are the 10 representative snapshots defined?
- Fig. 6b: Both strong and weak binder are explained with nM Kd in brackets. Is this a mistake?

Reviewer #2

(Remarks to the Author)
Allain and coworkers investigate the biophysical basis for anti cooperativity in binding of the bacterial RsmE protein dimer to repeated GGA RNA signals within SL2 of the bacterial RsmZ nc RNA. Binding of the second protein is 10-30 fold weaker compared to the first, and this could help in releasing RsmE from mRNAs to facilitate turnover.

The authors convincingly show that this occurs because the initial binding event increases conformation entropy for the second empty site, by partially unfolding tjhe C-terminal helix. Therefore, there is an increased entropic cost to pay when the second copy of the protein binds to the 'empty' site. There is a very nice correlation of thermodynamic data (entropy changes) from ITC, and NMR analysis of protein dynamics using backbone relaxation, and this makes the manuscript conclusions very convincing.

This is a very solid and well executed study. which should be of broader interest to other systems where anti-cooperativity is observed and where mechanistic conclusions might not be as clear.

The only criticism is the emphasis on Newton's cradle; I do not see the analogy to be so pertinent as to give it such prominence in the title etc; emphasis on anti cooperativity in the title would, I believe, better reflect what is in the manuscript and probably invite more readers.

Reviewer #3

(Remarks to the Author)
In their manuscript, Finol et al., investigate the mechanistic basis of negative cooperativity in RNA binding by the P. protegens translational repressor RsmE, a key component of the Csr/Rsm global regulatory system. This study provides mechanistic insight into how negative cooperativity arises in RNA-protein interactions, an underexplored aspect of regulatory networks. The authors present compelling evidence that high-affinity RNA binding at one site of the RsmE dimer reduces the binding affinity at the second site, and they explain how this affect regulatory function. Negative cooperativity is proposed to play a crucial role in enabling ncRNAs to sequester Rsme while still allowing its release, thereby supporting dynamic gene regulation. Using a rigorous combination of biophysical methods including NMR spectroscopy, ITC, and MD simulations, the authors systematically dissect the interaction between RsmE and its RNA ligand SL2. Their results support a model where RNA binding at one site structurally destabilizes the second, unbound site disfavoring RNA biding.

To understand the basis of this long-range communication, the authors examined the hydrogen bond network of the central beta-strands using heteronuclear NOE, HDX, and temperature coefficients of the NH groups. These experiments reveal that allosteric changes propagate through the anti-parallel beta-sheet network leading to a proposed “Newtons cradle” model where the binding of the RNA at one site transfers conformational entropy through the beta-sheets, destabilizing the second site. Overall, this work has broad implications for understanding how bacterial systems integrate environmental cues to regulate gene expression and offers a generalizable model for allosteric control in symmetric RNA-binding proteins.

The work is without question rigorous, substantial and will be of broad interest to the Nat. Comm. readership. However, I have some questions and made some suggestions that I think will improve the overall presentation of the manuscript:

1. The authors use sophisticated NMR approaches, however for probably the most interesting and novel section of their study, probing the dynamics of the exchange of information between the two binding sites, the authors seem to miss an opportunity to apply relaxation-based approaches to probe these dynamics. They present the heteronuclear NOE data, but why not measure T1 and T2 relaxation rates? I would expect that the allosteric network between the two binding sites would involve fast-timescale dynamics. While not in vogue, a model-free analysis of these data could lead to insights about the nature of the motions, and if it involves contributions from conformational and/or chemical exchange. With the hetNOE experiment, I would have expected these data (if not the associated analysis) to be included. Further, along these lines, the authors use Ca and Cb chemical shifts to assess the unfolding or dissolution secondary structure and to probe µs-ms dynamics. Were relaxation dispersion or chemical exchange methods, an approach that in my opinion seems tailor-made for this system, considered for this project?

2. On line 204 the authors state “…additional fast timescale backbone motion was also detected in some residues of the bound beta3 and beta4…” This statement is not supported by the data presented in either Fig. 4b or c.

3. For the observed line broadening, how can the authors be sure that transient associations rather than solely conformational deformation (unfolding) contribute to the observed line broadening. Are they detecting mixed exchange processes that cannot easily be disentangled by simply measuring peak half-height (if that is what was done)?

4. It is possible I missed something, but how is one supposed to interpret a negative chemical shift perturbation as shown in

Supp. Fig 7? This is unclear since it may be confused with the delta carbon shifts (which report on secondary structure).

5. I appreciate the cleverness of the authors approach to identify and assign the resonances arising from the minor states, however, as I understand the approach as described, the signals from state III were assigned based on assumptions about state I and II, thus these are tentative assignments and should be marked as such.

6. They used MD to uncover the mechanism creating the high affinity site. Could these trajectories be analyzed to gain insight into the proposed allosteric mechanism?

Minor Comments:

7. A cartoon depicting the terminology apo, halo, semi-halo etc. would be a welcome addition to Figure 1. Also, the introduction, by calling Figure 1, does not seem separate or distinct from the results section. If Figure 1 is a repeat of previously published experiments, maybe it should be moved to the supplemental to help streamline the manuscript.

8. I looked carefully but could not find the definition of the abbreviation CTDR.

9. The authors use of NH-CS perturbations and NH-CSP, and CS, CS perturbations, and CSP is distracting. I suggest using CSP and chemical shifts to denote the different measurements.

10. Inconsistent use of capitalization (Sodium ion vs. sodium ion, Potassium phosphate etc.) is especially evident in the captions.

11. incorrect hyphenation e.g. 10-µs

12. Caption for Fig. 2d – 10 µS should be 10 µs

13. Generally, in many places, the text used in figure labels and axis labels are too small to be readily interpretable, some of the text is obscured by the figure, especially the residue labeling in some of the structures. (for example Figs. 2c, 2d, 3a, 5c, 6c etc.)

14. What are the details of the procedure used to extract the line broadening values? If measuring peak half-height was used, was a line shape simulation employed? Also, what criteria was used to judge if the change in line broadening between two conditions was significant?

15. In Fig. 5c it might be more clear to report the fitted exchange rate instead of the entire equation for each curve.

16. I do not understand the rationale for the extended text sections, Sections 1-3 seem like they are detailed methods, while section 4 could be considered an expanded theory (also appropriate for M and M).

Version 1:

Reviewer comments:

Reviewer #1

(Remarks to the Author)
I thank the authors for addressing my major and minor points to full satisfaction. And also for clarifying the issue with the missing fit of n in sequential ITC binding. This makes absolute sense.
This is a very nice piece of work and I higly recommend publication.

Reviewer #2

(Remarks to the Author)
As reviewer 2, I was already happy with the manuscript except the emphasis on Newton's cradle in the title. It is the author's manuscript, and therefore I respect their desire to choose whichever word they want to describe their work.

Reading the other reviewer's comments, though, points 1 and 3 of reviewer 3 made me think there is more to learn about protein dynamics, which is the reviewer's point.

The line broadening described by the authors suggest slower dynamics than can be measured by HetNOE or T1, but perhaps accessible with relaxation dispersion etc and perhaps with the low frequency component of T2. I know protein dynamics is not fashionable anymore, but this could be an interesting first year graduate student future project, if feasible.

I would also be a little cautious about dismissing association as a cause for line broadening; even if the off rate is slow, ruling out exchange broadening due to the complex dissociating, conformational heterogeneity within a binding site can cause line broadening. This is more often seen for lower affinity interactions but we have seen these situations even with low nM complexes.

My comments should not be seen as preventing publication. These are issues I thought about quite a bit in the past and the reviewer's comments triggered my memory.

Reviewer #3

(Remarks to the Author)
Authors were highly responsive to review suggestions. Article is suitable for publication.

Open Access This Peer Review File is licensed under a Creative Commons Attribution 4.0 International License, which permits use, sharing, adaptation, distribution and reproduction in any medium or format, as long as you give appropriate credit to the original author(s) and the source, provide a link to the Creative Commons license, and indicate if changes were made.
In cases where reviewers are anonymous, credit should be given to 'Anonymous Referee' and the source.
The images or other third party material in this Peer Review File are included in the article's Creative Commons license, unless indicated otherwise in a credit line to the material. If material is not included in the article's Creative Commons license and your intended use is not permitted by statutory regulation or exceeds the permitted use, you will need to obtain permission directly from the copyright holder.
To view a copy of this license, visit https://creativecommons.org/licenses/by/4.0/

REVIEWER COMMENTS are in Italicized and bold text.

Our response is in plain text.

Reviewer #1 (Remarks to the Author):

In the submitted manuscript, Finol and colleagues present a thorough NMR- and MD-based study targeting the molecular details of negative cooperativity in RsmZ RNA-binding by the dimeric RsmE protein. This process is utilized by bacteria, where the ncRNA RsmZ acts as a sponge to remove RsmE from mRNAs' SD sequences and thereby enabling translation of the mRNA. The effective hand-over of RsmE from its two unequal binding sites around the SD sequence to neighbouring stem-loops in RsmZ has remained incompletely understood, also in light of a relatively broad range of possible affinities between RsmE monomers and the respective GGA motifs in the mRNA or RsmZ.

The authors here use ITC together with NMR CSP, NOESY and relaxation data and MD simulations to suggest a model (in analogy to Newton's cradle), in which they suggest that changes in conformational entropy along the two binding sites in the RsmE dimer account for the modulation of affinity for RNA in the stepwise binding process. This is solidly corroborated by relaxation and H/D exchange data, guided by MD simulations suggesting multiple bound states.

The manuscript addresses a non-trivial question and uses very detailed insights into a protein-RNA system, which is quite well understood on the structural side, while mechanistic details have remained incompletely explored. The presented data are in principle trustworthy, well-presented and complementary, i.e. they yield a robust set of results to feed the mechanistic model. The text is also well written, and the manuscript overall concise and straightforward.

I feel the story does deserve to be published in this journal, as it manages to yield insight into fine details for how cooperativity works in this way, something that may also help to push similar systems by the choice of proper methods. There is little I have to criticize about the given data, but I would need a bit of add-on to support the model with suitable experimental data. I think this will strengthen the arguments and make the story be rounded up.

These are my concrete points:

- Regarding the model: In what is shown in Fig. 6 binding of RsmZ SL3 competes with binding of the RsmE subunit to the SD SL motif within the mRNA. From the previously published numbers this appears to be not so likely. I find the suggested model justified, but I wonder if the authors can provide experimental evidence supporting this mechanism, e.g. in designing a competing binding experiment with the relevant players? Can the effect of SL2-binding towards the affinity for the SD SL in the other monomer be shown or quantified? How much would this be decreased to allow unbinding?

We thank Reviewer#1 for the positive assessment of our work and the insightful suggestion. It has allowed us to strengthen this aspect of our study and to provide further evidence on the functional role of the anti-cooperativity in RsmE dimer. In this regard, we have performed a series of experiments to assess the effect of SL2 RNA binding on hcnA mRNA containing an SD motif at the bound site. A short text was added to the main text in **lines 72-82** together with additional panels (**d-e**) in **Fig.1**. It shows NMR evidence for allostery affecting the bound SD RNA when SL2 RNA binds the second site. In addition, a new Supplementary Text 1 and Supplementary Fig.1 show the affinity change caused by SL2 binding, and provide more detailed results and explanatory text. The affinity of SD changes from ~10nM when singly bound to RsmE dimer to ~300nM when SL2 is bound at the second site, a 30-fold reduction in affinity. Together with the avidity effect of SL3 being linked to SL2 in RsmZ RNA, we believe this to be sufficient to efficiently displace RsmE from the SD site.

- Also, the authors claim there is no cooperativity with only weak binders. Is this supported by experimental data, e.g. by H/D exchange or unfolding studies in response to RsmZ SL3?

Yes, it is supported. Our previous investigation on the different RsmE-binding RNAs from RsmZ showed that weak binders such as SL3, when titrated into RsmE-containing solutions using ITC, didn't show two binding events and the $^{1}H^{15}N$-HSQC spectra from the same titrations showed a simple two-state transition from free to bound, rather than showing intermediates states. These observations are reported in these two articles (**references 7 & 8**):

Duss, O., Michel, E., Diarra dit Konté, N., Schubert, M. & Allain, F. H.-T. Molecular basis for the wide range of affinity found in Csr/Rsm protein–RNA recognition. Nucleic Acids Res. 42, 5332–5346 (2014).

Duss, O. et al. Structural basis of the non-coding RNA RsmZ acting as a protein sponge. Nature 509, 588–592 (2014).

and they are referenced in the discussion of weak binders (starting in **line 372**).

- Adding to the above: The data on cooperativity have been obtained with SL2 as the readout, which makes sense. However, in the model the relevant relative affinities and thus effects apparently have to take into consideration initial one-site saturation with SL2, and then SL3! Are concentrations of RsmE and RsmZ in the cell known? Could an alternative model be, that two RsmZ RNAs remove RsmE as a dimer through their individual SL2s?

To our knowledge, the concentration of these two biomolecules and their stoichiometry in cells remains unknown, although several studies, which aimed to explore changes in their abundance upon diverse stimuli, have assessed their relative levels using non-quantitative methods such as Western and Northern blots.

The proposed model (of two SL2 from two different RsmZ RNAs binding on RsmE) is indeed a possible alternative mechanism. However, we believe that the local concentration effect of having SL3 within the same RNA molecule with SL2 in RsmZ is more likely to have an avidity effect with a higher effective affinity than two

independent SL2 RNAs. This is consistent with our previous work showing that at 1:1 ratio with RsmZ RNA containing both SL2 and SL3, we see no evidence that two copies of SL2 are bound. Rather, one site is bound with SL2 and the other with SL3.

Duss, O. et al. Structural basis of the non-coding RNA RsmZ acting as a protein sponge. Nature 509, 588–592 (2014).

ITC related comments:

Major comment:

- All experiments assume that a SL2-RsmE dimer ratio of 1:1 leads to exclusive saturation of the high-affinity monomer. But seeing the numbers from ITC there should be a mentionable amount of two-site binding (statistically). I do not question the numbers obtained from experiments, but I suggest to mention this in the text, and ideally quantify (at least theoretically) all given states in the semi-holo complex.

This is a good point. While the reported Kd1 and Kd2 values would theoretically result in observable signals of apo and holo states at 1:1 ratio, such signals were not detected. This discrepancy may be associated to the inability of ITC measurements to differentiate Kd values under 1nM. Thus, the measured Kd1 value of 1.4nM is likely to be an upper bound on the actual binding constant, which could be much lower. This would increase the negative cooperativity and result in much smaller populations of the *apo* and *holo* states. We have now added additional text on **lines 87-94**, explaining this.

Minor comment:

- ***ITC data should be shown with all repetitions and error bars be added to the plots of thermodynamic parameters (e.g. Fig. 1a). Also, n-values should be given (molar ratios).***

We thank Reviewer#1 from spotting the lack of error bars in our plots, we have added them to the plots and we have also included the data from the repetitions in supplementary table 1.

Regarding the two-site binding and the n-values, when fitting the ITC spike integrals to the sequential binding model, the n-values are defined by default to two sites and during the best fit determination, there is therefore no fitting of the N parameter. To further explain this aspect of the ITC data fitting, herein, we included an extract on this topic from the ITC Data analysis manual from MicroCal, the initial manufacturer of the ITC device, now Malvern paranalytical.

> "The model of Sequential Binding Sites assumes a fixed sequence of binding, i.e., the first ligand which binds to an individual molecule always binds to site 1, the second ligand which binds to an individual molecule always binds to site 2, etc. The number of sequential sites must be exactly integral (1,2,3,…) so there is no fitting parameter equivalent to N, and best-

fit is determined by only two parameters, K and H, at each site once the total number of sites has been selected by the operator."

This manual can be found online and the extract is from page #65, here:

https://bif.wisc.edu/wp-content/uploads/sites/389/2017/11/ITC_Data_Analysis_in_Origin.pdf

We have also included some text to explain this on **line 521**.

- ***Just for a discussion: could there also be cooperative effects within the RNA SLs of RsmZ?***

Yes, there is a cooperative effect in RsmZ-RsmE interactions. We have summarized it in a short sentence in **lines 52-54** in the introduction:

"*Since all the isolated binding sites have micromolar or lower dissociation constants, RsmZ sequesters RsmE dimers in a sequential and cooperative manner*".

This is one of the main conclusions from these two articles:

Duss, O., Michel, E., Diarra dit Konté, N., Schubert, M. & Allain, F. H.-T. Molecular basis for the wide range of affinity found in Csr/Rsm protein–RNA recognition. Nucleic Acids Res. 42, 5332–5346 (2014).

Duss, O. et al. Structural basis of the non-coding RNA RsmZ acting as a protein sponge. Nature 509, 588–592 (2014).

As minor issues:

- ***Please provide the comparison of spectral overlays showing SL2 vs. 4bpSL2 titrations!***

We thank Reviewer#1 for this comment; for completeness, we have now included the 1H15N-HSQC spectral overlays in the **Supplementary Fig.8**. which is referenced in **lines 189-192** of the text when discussing the NH-CSP in the SL2 and 4bpSL2-RsmE dimer complexes.

- ***Page 5, Fig. 2e: how exactly are the 10 representative snapshots defined?***

These 10 representative snapshots were derived from the frames in the MD simulations that matched the two maximum peaks that were observed in the 2D-plot correlating the distance of Ade26(H2)–B-I47(Hγ2) versus Ade(H2)–B-A57(Hβ) distances. For bound state I, we retrieved all frames that showed Ade26(H2)–B-I47(Hγ2) distances between 4.8 and 4.9 Å and Ade(H2)–B-A57(Hβ) distances between 2.9 and 3.0 Å. For bound state II, we retrieved all the frames that showed Ade26(H2)–B-I47(Hγ2) distances between 5.0 and 5.1 Å and Ade(H2)–B-A57(Hβ) distances between 11.0 and 11.1 Å. In both cases, a few thousand frames were retrieved, and 10 representative frames were randomly selected, their Cα-backbone were aligned and these are displayed in **Figure 2e**.

We have now included these details in the methods section in **lines 544-553**.

- Fig. 6b: Both strong and weak binder are explained with nM Kd in brackets. Is this a mistake?

We thank Reviewer#1 for identifying this typo, we have corrected it in the figure.

Reviewer #2 (Remarks to the Author):

Allain and coworkers investigate the biophysical basis for anti-cooperativity in binding of the bacterial RsmE protein dimer to repeated GGA RNA signals within SL2 of the bacterial RsmZ nc RNA. Binding of the second protein is 10-30 fold weaker compared to the first, and this could help in releasing RsmE from mRNAs to facilitate turnover.

The authors convincingly show that this occurs because the initial binding event increases conformation entropy for the second empty site, by partially unfolding the C-terminal helix. Therefore, there is an increased entropic cost to pay when the second copy of the protein binds to the 'empty' site. There is a very nice correlation of thermodynamic data (entropy changes) from ITC, and NMR analysis of protein dynamics using backbone relaxation, and this makes the manuscript conclusions very convincing.

This is a very solid and well executed study. which should be of broader interest to other systems where anti-cooperativity is observed and where mechanistic conclusions might not be as clear.

The only criticism is the emphasis on Newton's cradle; I do not see the analogy to be so pertinent as to give it such prominence in the title etc; emphasis on anti cooperativity in the title would, I believe, better reflect what is in the manuscript and probably invite more readers.

We thank Reviewer#2 for the positive assessment of our work. We have used Newton’s cradle as an easy-to-understand analogy to explain the transmission of conformational entropy between the two sites which facilitates RNA handover. This is encapsulated in the final figure of the paper which summarizes our mechanistic model. In addition, the manuscript has been issued as a preprint with the current title. For these reasons we prefer to keep our title unchanged. We have put “anti cooperativity” in the keywords and negative cooperativity occurs in the abstract and hope this is acceptable.

Reviewer #3 (Remarks to the Author):

In their manuscript, Finol et al., investigate the mechanistic basis of negative cooperativity in RNA binding by the P. Protegens translational repressor RsmE, a key component of the Csr/Rsm global regulatory system. This study provides

mechanistic insight into how negative cooperativity arises in RNA-protein interactions, an underexplored aspect of regulatory networks. The authors present compelling evidence that high-affinity RNA binding at one site of the RsmE dimer reduces the binding affinity at the second site, and they explain how this affect regulatory function. Negative cooperativity is proposed to play a crucial role in enabling ncRNAs to sequester Rsme while still allowing its release, thereby supporting dynamic gene regulation. Using a rigorous combination of biophysical methods including NMR spectroscopy, ITC, and MD simulations, the authors systematically dissect the interaction between RsmE and its RNA ligand SL2. Their results support a model where RNA binding at one site structurally destabilizes the second, unbound site disfavoring RNA binding.

To understand the basis of this long-range communication, the authors examined the hydrogen bond network of the central beta-strands using heteronuclear NOE, HDX, and temperature coefficients of the NH groups. These experiments reveal that allosteric changes propagate through the anti-parallel beta-sheet network leading to a proposed "Newtons cradle" model where the binding of the RNA at one site transfers conformational entropy through the beta-sheets, destabilizing the second site. Overall, this work has broad implications for understanding how bacterial systems integrate environmental cues to regulate gene expression and offers a generalizable model for allosteric control in symmetric RNA-binding proteins.

The work is without question rigorous, substantial and will be of broad interest to the Nat. Comm. Readership. However, I have some questions and made some suggestions that I think will improve the overall presentation of the manuscript:

1. The authors use sophisticated NMR approaches, however for probably the most interesting and novel section of their study, probing the dynamics of the exchange of information between the two binding sites, the authors seem to miss an opportunity to apply relaxation-based approaches to probe these dynamics. They present the heteronuclear NOE data, but why not measure T1 and T2 relaxation rates? I would expect that the allosteric network between the two binding sites would involve fast-timescale dynamics. While not in vogue, a model-free analysis of these data could lead to insights about the nature of the motions, and if it involves contributions from conformational and/or chemical exchange. With the hetNOE experiment, I would have expected these data (if not the associated analysis) to be included. Further, along these lines, the authors use Ca and Cb chemical shifts to assess the unfolding or dissolution secondary structure and to probe µs-ms dynamics. Were relaxation dispersion or chemical exchange methods, an approach that in my opinion seems tailor-made for this system, considered for this project?

We thank Reviewer#3 for the suggestion to include T1 and T2 data and a quantitative analysis of dynamics. We have indeed attempted such measurements. Unfortunately, due to the extensive line broadening in the 15N,1H HSQC spectra, quantitative analysis using e.g. the modelfree formalism was not possible. This is why we opted for a semi quantitative analysis of the line broadening in the HSQC spectra for slow timescale dynamics and used the hetNOE experiment to assess the sub-TauC motions.

2. On line 204 the authors state "…additional fast timescale backbone motion was also detected in some residues of the bound beta3 and beta4…" This statement is not supported by the data presented in either Fig. 4b or c.

We have dropped this statement since the differences are close to the range of the error bars.

3. For the observed line broadening, how can the authors be sure that transient associations rather than solely conformational deformation (unfolding) contribute to the observed line broadening. Are they detecting mixed exchange processes that cannot easily be disentangled by simply measuring peak half-height (if that is what was done)?.

Although transient association can contribute to line-broadening, we found it unlikely to significantly contribute to the observe line-broadening in the semi-holo complex at equilibrium. Due to the high affinity of SL2 for RsmE, the dissociation rate of SL2 is very slow. We generally observe that the signals of the semi-holo state are in slow exchange and distinguishable from both the apo and holo signals. Therefore, we do not believe the observed line broadening is due to the association/dissociation equilibrium.

Further details on the line broadening analysis can be found in our response to Reviewer#3, question 14, which is focused on this topic.

4. It is possible I missed something, but how is one supposed to interpret a negative chemical shift perturbation as shown in Supp. Fig 7? This is unclear since it may be confused with the delta carbon shifts (which report on secondary structure).

In **Suppl. Fig.7** (now **Supplementary Fig.8**), we reported the change in the NH-CSP in the side chain amides by subtracting the NH-CSP caused by SL2 binding from the NH-CSP caused by 4bpSL2 binding. Thus, the ∆NH-CSP shown in the figure is calculated using the following equation:

∆NH-CSP= 4bpSL2-induced NH-CSP – SL2-induced NH-CSP

This equation is now provided in the figure caption and the definition of CSP is provided in the methods section.

A negative ∆NH-CSP would indicate that the binding of SL2 to RsmE leads to larger NH-CSP in this side chain signal than the binding of 4bpSL2.

5. I appreciate the cleverness of the authors approach to identify and assign the resonances arising from the minor states, however, as I understand the approach as described, the signals from state III were assigned based on assumptions about state I and II, thus these are tentative assignments and should be marked as such.

We now indicate that the assignments of state III signals are tentative.

Main text, **line 101-103**: "and, due to the limited information obtainable, we consider the chemical shift assignment of state III as tentative".

6. They used MD to uncover the mechanism creating the high affinity site. Could these trajectories be analyzed to gain insight into the proposed allosteric mechanism?

For the revised manuscript, we have performed further analysis of the apo, semi-*holo* and *holo* MD simulations. The MD simulations show increased dynamic fluctuations particularly in β5 and the α-helix of the empty site for the semi-holo state. In addition, H-bond partner lengths and fluctuations increase for β2–β5 and β5–α-helix interactions. On the other hand, we don't observe changes for the remaining β-strand interactions. This may be due to the fact that simulations start from the coordinates of the holo complex and the timescale of the conformational changes transmitted to the empty site are not probed sufficiently. We have included a brief statement on the in-silico observations in the main manuscript **lines 221-229**, we have also included more detailed explanations in a **Supplementary Text 6**. The in-silico data are now provided in a new **Supplementary figure 12**. The corresponding methodologies were added to the methods section "Molecular Dynamics Simulations".

Minor Comments:

7. A cartoon depicting the terminology apo, halo, semi-halo etc. would be a welcome addition to Figure 1. Also, the introduction, by calling Figure 1, does not seem separate or distinct from the results section. If Figure 1 is a repeat of previously published experiments, maybe it should be moved to the supplemental to help streamline the manuscript.

This is a good suggestion. We have included an explanatory cartoon in Figure 1 to ease the understanding of the data.

Although the existence of negative allostery was previously reported, the assignments of the apo and semi-holo chemical shifts and the quantification of backbone NH-CSP are reported for the first time in **Figure 1** in this manuscript. We have also included two more panels in **Fig.1** to further illustrate the existence and function of the negative allostery in RsmE dimer.

8. I looked carefully but could not find the definition of the abbreviation CTDR.

We thank Reviewer#3 for spotting this issue. The above-mentioned abbreviation could be found in the subheading (**lines 84-85**), but it had typo. It has been corrected.

9. The authors use of NH-CS perturbations and NH-CSP, and CS, CS perturbations, and CSP is distracting. I suggest using CSP and chemical shifts to denote the different measurements.

We have harmonized and simplified the use of these abbreviations throughout the manuscript.

10. Inconsistent use of capitalization (Sodium ion vs. sodium ion, Potassium phosphate etc.) is especially evident in the captions.
11. incorrect hyphenation e.g. 10-µs
12. Caption for Fig. 2d – 10 µS should be 10 µs
We thank Reviewer#3 for the great care taken to spot these details. We have fixed these issues throughout the manuscript.

13. Generally, in many places, the text used in figure labels and axis labels are too small to be readily interpretable, some of the text is obscured by the figure, especially the residue labeling in some of the structures. (for example Figs. 2c, 2d, 3a, 5c, 6c etc.)

We agree and have increased the font size for the text in most of the figures.

14. What are the details of the procedure used to extract the line broadening values? If measuring peak half-height was used, was a line shape simulation employed? Also, what criteria was used to judge if the change in line broadening between two conditions was significant?

We thank Reviewer#3 for spotting this omission; we had forgotten to include this segment of the methods section in the submitted manuscript. You can now find it in the resubmitted version in **lines 476-489**. Briefly, due to the limited signal-to-noise, particularly for the line-broadened signal in the empty site, lineshape fitting was not possible in many cases. We therefore opted for a qualitative assessment of linewidth changes. We classified peaks into categories: sharp, broad, and near to noise based on their signal to noise and whether their lineshape could be fitted. Changes in category are represented by spheres of different diameters in the figure. We have also modified the figure caption 4a to read "Qualitative mapping of line-broadening".

15. In Fig. 5c it might be more clear to report the fitted exchange rate instead of the entire equation for each curve.

We agree, and now report the exchange rate (*k*) only. We have also applied this improvement to the corresponding **supplementary Fig. 15**.

16. I do not understand the rationale for the extended text sections, Sections 1-3 seem like they are detailed methods, while section 4 could be considered an expanded theory (also appropriate for M and M).

We understand your concern. However, considering that Nature Communications is a broad scope journal, we have included these sections to give additional details and rationale for our reasoning which might not be immediately clear to readers in other fields without further extending the word count of the main text.
